# A cell-of-origin epigenetic tracer reveals clinically distinct subtypes of high-grade serous ovarian cancer

**DOI:** 10.1186/s13073-020-00786-7

**Published:** 2020-10-30

**Authors:** Pietro Lo Riso, Carlo Emanuele Villa, Gilles Gasparoni, Andrea Vingiani, Raffaele Luongo, Anna Manfredi, Annemarie Jungmann, Alessia Bertolotti, Francesca Borgo, Annalisa Garbi, Michela Lupia, Pasquale Laise, Vivek Das, Giancarlo Pruneri, Giuseppe Viale, Nicoletta Colombo, Teresa Manzo, Luigi Nezi, Ugo Cavallaro, Davide Cacchiarelli, Jörn Walter, Giuseppe Testa

**Affiliations:** 1 Department of Experimental Oncology, IEO, European Institute of Oncology IRCSS, Milan, Italy; 2 Department of Genetics, University of Saarland, Saarbrücken, Germany; 3 Department of Pathology, Biobank for Translational Medicine Unit, IEO, European Institute of Oncology IRCSS, Milan, Italy; 4 Present affiliation: Department of Pathology, Fondazione IRCSS Istituto Nazionale Tumori, Milan, Italy; 5 Department of Oncology and Hemato-Oncology, University of Milan, Milan, Italy; 6 SEMM, European School of Molecular Medicine, Milan, Italy; 7 Telethon Institute of Genetics and Medicine (TIGEM), Armenise/Harvard Laboratory of Integrative Genomics, Pozzuoli, Italy; 8 Division of Gynecologic Oncology, IEO, European Institute of Oncology IRCSS, Milan, Italy; 9 Unit of Gynecological Oncology Research, IEO, European Institute of Oncology IRCSS, Milan, Italy; 10 Present affiliation: DarwinHealth Inc., New York, NY, USA; 11 Novo Nordisk Research Center Seattle, Inc. (NNRCSI), Seattle, WA USA; 12 Department of Translational Medicine, University of Naples Federico II, Naples, Italy

## Abstract

**Background:**

High-grade serous ovarian cancer (HGSOC) is a major unmet need in oncology. The remaining uncertainty on its originating tissue has hampered the discovery of molecular oncogenic pathways and the development of effective therapies.

**Methods:**

We used an approach based on the retention in tumors of a DNA methylation trace (OriPrint) that distinguishes the two putative tissues of origin of HGSOC, the fimbrial (FI) and ovarian surface epithelia (OSE), to stratify HGSOC by several clustering methods, both linear and non-linear. The identified tumor subtypes (FI-like and OSE-like HGSOC) were investigated at the RNAseq level to stratify an in-house cohort of macrodissected HGSOC FFPE samples to derive overall and disease-free survival and identify specific transcriptional alterations of the two tumor subtypes, both by classical differential expression and weighted correlation network analysis. We translated our strategy to published datasets and verified the co-occurrence of previously described molecular classification of HGSOC. We performed cytokine analysis coupled to immune phenotyping to verify alterations in the immune compartment associated with HGSOC. We identified genes that are both differentially expressed and methylated in the two tumor subtypes, concentrating on PAX8 as a *bona fide* marker of FI-like HGSOC.

**Results:**

We show that:

- OriPrint is a robust DNA methylation tracer that exposes the tissue of origin of HGSOC.

- The tissue of origin of HGSOC is the main determinant of DNA methylation variance in HGSOC.

- The tissue of origin is a prognostic factor for HGSOC patients.

- FI-like and OSE-like HGSOC are endowed with specific transcriptional alterations that impact patients’ prognosis.

- OSE-like tumors present a more invasive and immunomodulatory phenotype, compatible with its worse prognostic impact.

- Among genes that are differentially expressed and regulated in FI-like and OSE-like HGSOC, PAX8 is a *bona fide* marker of FI-like tumors.

**Conclusions:**

Through an integrated approach, our work demonstrates that both FI and OSE are possible origins for human HGSOC, whose derived subtypes are both molecularly and clinically distinct. These results will help define a new roadmap towards rational, subtype-specific therapeutic inroads and improved patients’ care.

## Background

Ovarian cancer is the 8th most common cause of cancer death in women worldwide, being the 18th most common cancer worldwide and accounting for more than 200,000 new cases every year [1]. Its most prevalent form is high-grade serous ovarian cancer (HGSOC) which accounts for more than 70% of cases and is usually diagnosed at already advanced stages being mostly asymptomatic earlier on [2]. In the 30 years since the introduction of carboplatin-based regimens, the cure rate has changed only negligibly, largely due to the precocious dissemination (favored by the anatomic continuity with the abdominal cavity) and the limited number of physiopathologically meaningful models that can recapitulate the pathogenesis and progression of the human disease, an increasingly recognized need for the field as a prerequisite for the identification and validation of new therapeutic targets [3, 4]. In turn, the lack of actionable human models is due to a significant extent to the persistent uncertainty regarding the cell of origin of HGSOC, with the two candidate originating tissues identified in the distal tract of the fallopian tube (fimbrial epithelium, FI) and the epithelial lining of the ovary (ovarian surface epithelium, OSE) [5]. In recent years, growing evidence has pointed to the FI as the main source of HGSOC, starting from prophylactic salpingo-oophorectomy specimens from patients with increased risk of ovarian cancer, which revealed how the fimbria is frequently affected by pre-cancerous lesions referred to as serous tubal intraepithelial neoplasia (STIC). This suggested that lesions labeled as ovarian tumors could actually result from seeding of primary fimbrial tumors, through trapping of STIC-derived cells in the lumen of the ovary as inclusion cysts, favored by the ruptured stigmas of the surface epithelium during the menstrual cycle. Initial transcriptomic and methylomic analyses also uncovered higher similarity between HGSOC and FI with respect to the OSE, supporting a non-primarily ovarian origin [6, 7]. Mutational analysis of STIC, primary HGSOC, and peritoneal metastasis from the same patient revealed the presence of a shared mutational spectrum, thus reinforcing the notion of a primary if not exclusive fallopian origin [8]. These lines of evidence notwithstanding, other convergent sources failed to settle this fundamental developmental question, starting from the observation that in a significant proportion of HGSOC samples no STIC precursor lesion can be identified. This indicates that the fimbrial origin seeding model cannot be universally applied to all HGSOC samples. Indeed, the reverse route of dissemination has been also shown to be equally plausible, with genomic studies supporting the possibility that at least a fraction of STIC actually represent metastatic lesions of primarily ovarian lesions, a model underscored by the experimental finding that HGSOC-derived spheroids can implant into the fallopian tube epithelium [9]. While it is yet to be clarified whether also early OSE lesions can seed onto the fimbrial surface and give rise to STIC, there thus remains fundamental uncertainty about the developmental origin of HGSOC, both in terms of the general distribution between FI or OSE origins and, more importantly, in terms of patient-tailored assays to assign developmental origin on a case-to-case basis.

DNA methylation is the best characterized epigenetic mark, whose tightly regulated deposition and propagation at specific loci ensures the stable inheritance of gene expression responses across cell division. Consistent with the key relevance of this epigenetic regulation, mutations and/or dysregulation in the DNA methylation machinery are a well-established feature of tumorigenesis [10]. Yet, despite such extensive aberrations, accumulating evidence has shown that a subset of the original DNA methylation prints can be retained during tumor evolution, akin to the tissue-specific traces that are acquired and maintained throughout development [11]. Indeed, analyzing the DNA methylome of tumor cells has allowed to track tumor clonal evolution, to an extent comparable to genetic fingerprinting. Importantly, the retention in tumor cells of epigenetic prints of the tissue of origin has also allowed to associate the tissue of origin to cancers of unknown primary (CUP) [12], affording for the first time a tailored treatment and improved care for patients. Also, the definition of tumor-specific DNA methylation traits allowed to re-classify central nervous system tumors, impacting their routine diagnostics for these diseases [13].

Here we present a novel approach to solve the attribution of the tissue of origin for HGSOC, grounded in the tumor retention of a cell of origin-specific DNA methylation print (OriPrint) that allows to robustly stratify HGSOC in FI- and OSE-originated tumors (FI-like and OSE-like) robustly across all available datasets. We show that both epithelia serve as *bona fide* origin for this disease and that the cell of origin explains most of the variance existing among tumors. We then translate these findings into a transcriptional readout through a particularly cost-effective, clinically relevant transcriptomic analysis. This revealed the prognostic value of a cell of origin-based classifier in an independent, well-characterized retrospective cohort. Specifically, we found that OSE-like tumors carry a significantly worse prognosis that can be ascribed to a reduced inflammatory response, coupled to increased survival and cell-to-cell signaling in this specific tumor subset. Moreover, we apply our stratification strategy to published datasets, finding a consistent enrichment for the mesenchymal molecular subtype in OSE-like tumors, which explains their worse prognosis. Finally, our deconvolution of developmental origin uncovers new genes and pathways specific for OSE-like HGSOC that open new strategies for an improved management of HGSOC patients.

## Methods

### Primary cells

For all samples, the diagnosis of high-grade serous carcinoma was confirmed by pathology review according to the 2014 World Health Organization (WHO) classification (Kurman). High-grade serous epithelial OC cells were derived from peritoneal ascites (AS samples) or from tumor biopsies of patients (HGSOC) who had primary, non-recurrent OC and had not yet undergone chemotherapy. Primary cells from FI and OSE were derived from non-neoplastic fimbriae and ovaries, respectively, from patients undergoing hystero-salpingo-oophorectomy being affected by myomas (large and/or multiple symptomatic leiomyomas) or cervical neoplasms (mainly squamous cell carcinoma of the uterine cervix). The isolation and culture of primary cells were performed as described previously [14]. More in detail, primary tumor samples were cut into small pieces using a scalpel and then minced with scissors. Samples were then digested for 1 to 4 h (depending on samples) with Epithelia Digestion Medium (EDM) composed of Ham’s F12 (Gibco, #31765035) and DMEM (Sigma, #D6429) 1:1, supplemented with 1% penicillin/streptomycin (Gibco, #15070063), 1 μg/ml insulin (Sigma, #I0516), 0.2 μg/ml hydrocortisone (Sigma, #H0888), 10 ng/ml EGF (Peprotech, #100-15), 200 U/ml collagenase (Sigma, #C2674), and 100 U/ml hyaluronydase (Sigma, #H3884). The digested tissue suspensions were passed on a 40-μm cell strainer and further sheared passing them through a P1000 tip and centrifuged at 500*g* for 3 min. In case of a visible red pellet, erythrocytes were lysed by resuspending the cell pellet in ACK buffer (Gibco, #A1049201) for 3 min at room temperature. Cells were then washed in PBS and centrifuged at 500*g* for 3 min and plated on Collagen I-coated flasks (BD Biosciences). For metastatic ascites, the used protocol is the same, substituting the digestion steps with a centrifuge at 500*g* for 3 min. Normal fimbria and ovary tissues were incubated in 1 mg/ml Dispase (Stem Cell Technologies, #07913) for 30′ at 37 °C. Then, epithelial cells from the distal portion of the fimbria and the surface of the ovary were scraped with a scalpel and pelleted at 500*g* for 3 min, red blood cells were lysed by ACK solution, and derived-epithelial cells were plated as for solid tumors.

Cells derived from all tissue sources were maintained in culture with an epithelial-specific culture medium (EPI medium) which is composed of a 1:1 mixture of DMEM-F12, supplemented with 1% FBS NA (ThermoFisher, #26140), 1% Pen-Strep, 0.2% of gentamycin (Lonza, #17-519L), 0.2% amphotericin (Roche, #15290026), 10 mM HEPES pH 7.5 (Gibco, #11560496), 10 μg/ml human transferrin (Sigma, #T8158), 1 μg/ml insulin, 1 μg/ml hydrocortisone, 50 μM l-ascorbic acid (Sigma, #A4544), 15 nM sodium selenite (Sigma, #S5261), 0.1 mM ethanolamine (Sigma, #E9508), 50 ng/ml cholera toxin (Sigma, # C8052), 10 ng/ml EGF, 35 μg/ml BPE (Gibco, 13028014), 10 nM T3 (Sigma, #T5516), and 10 nM β-estradiol (Sigma, #E2758). Cells were used at passages 3 to 5.

### Microarray processing and DNA methylation analyses

gDNA from cells was extracted by the DNeasy Blood and Tissue kit (Qiagen) according to the manufacturer’s instructions. For each sample, 500 ng of genomic DNA was bisulfite converted using the EZ-DNA methylation Gold Kit (Zymo research) according to the kit’s manual, except that the final elution volume was reduced to 12 μl. Per sample, 4 μl of bisulfite-converted DNA was used in either the Infinium Human Methylation 450k or the Infinium Methylation EPIC array (both Illumina) procedure according to the vendor’s protocol. Arrays were hybridized according to the manufacturer’s description and scanned on a HiScan system (Illumina). Idat files from the IEO cohort and the published datasets (Omaha [7, 15] and Melbourne [16, 17] cohorts) were processed using the minfi R package [18] (1.26.0). 450k and EPIC arrays were combined through the combineArrays command (minfi) and preprocessed through SWAN normalization. The three datasets *M* values were batch-corrected through ComBat from the SVA package, defining as batch the three datasets and modeling the matrix around the sample types (FI, OSE, HGSOC). To map CpGs to functional elements we used the RnBeads package [19]. To define OriPrint [20], differential methylation analyses were performed through the limma package [21] using adj. *P*.value < 0.05 and logFC > 1 as threshold.

Beta values (logit transformation of *M* values) were used for the following analyses. For Pearson’s correlation-based clustering, a distance matrix based on Pearson’s correlation was computed and clustering was performed using the hclust command, using ward.D2 as agglomeration method.

Beta values were imported in Python as anndata object, and we used SCANPY vs 1.3.1 [22] to plot UMAP and diffusion map for the data. For visualization purposes, we used 20 *n_neighbors*.

Pseudotime analyses were performed with SCANPY, and the pseudotime origin elements were selected based on their peripheral localization in the multiple dimensionality reduction graphs.

Louvain clustering [23] was calculated on the first 50 principal components, imposing (1) minimum number of elements or (2) agglomerating the resulting clusters, with consistent results. HDBScan [24] was performed on diffusion map’s coordinates, imposing the number of resulting clusters. Gaussian Mixture Model (GMM) clustering was performed with the scikit-learn module, on the first two principal components of variance defined by OriPrint, imposing the number of Gaussians and considering the best fitter (log-likelihood) out of 2000 iterations with multiple random initializations.

### Targeted bisulfite sequencing

Typically, 500 ng of genomic DNA was bisulfite converted using the EZ-DNA methylation Gold Kit (Zymo research) according to the kit’s manual. For PCR amplicons, locus-specific primers (Table 1) were designed with an in-house tool. PCRs were set-up in 30-μl reactions using 3 μl of 10x Hot Fire Pol Buffer (Solis BioDyne), 4 μl of 10 mM dNTPs (Fisher Scientific), 2.25 μl of 25 mM MgCl2 (Solis BioDyne), 0.6 μl of amplicon-specific forward and reverse primer (10 μM each), 0.3 μl of Hot FirePol DNA Polymerase (5 U/μl; Solis Biodyne), 1 μl of bisulfite-converted DNA, and 18.25 μl of double distilled water. PCRs were run in an ABI Veriti thermo-cycler (Thermo Fisher) using the following program: 95 °C for 10 min, then 40 cycles of 95 °C for 1 min, 2.5 min of 56 °C and 40 s at 72 °C, followed by 7 min of 72 °C and hold at 4 °C. PCR products were cleaned up using Agencourt AMPure XP Beads (Beckman Coulter). All amplified products were diluted to 4 nM, and NGS tags were finalized by a second PCR step (5 cycles) followed by a final clean-up (Agencourt AMPure XP Beads). Finally, all samples (set to 10 nM) were pooled, loaded on an Illumina MiSeq sequencing machine, and sequenced for 2 × 300 bp paired-end with a MiSeq reagent kit V3 (Illumina) to ca. 10 k–20 k fold coverage. The raw data was quality checked using FastQC and trimmed for adaptors or low-quality bases using the tools cutadapt and Trim Galore!. Paired reads were joined with the FLASh tool. Next, reads were sorted in a two-step procedure by (i) the NGS barcode adaptors to assign sample ID and (ii) the initial 15 bp to assign amplicon ID. Subsequently, the sorted data was loaded into the BioAnalyzer HT using the following settings: *analyzed methylation context* was set to “C,” *minimal sequence identity* was set to 0.9, and *minimal conversion rate* was set to 0.95. The filtered high-quality reads were then used for methylation calls of the respective CpGs.

**Table 1:** Primer sequences used in this study. “X” refers to sample-specific barcode sequences

	Forward primer (5′–3′)	Reverse primer (5′–3′)	Mapping (hg19)
PAX8 region	GTTTAATTTGGGAGGGAAAAGGTTGTT	AATTAAAACTCAACTACCTCCCTCTTC	chr2:114036300-114036716
PCR primer tags	TCTTTCCCTACACGACGCTCTTCCGATCT	GTGACTGGAGTTCAGACGTGTGCTCTTCCGATCT	
2nd PCR primers	CAAGCAGAAGACGGCATACGAGATXXXXXXGTGACTGGAGTTCAGACGTGTGCTCTTCCGATCT	AATGATACGGCGACCACCGAGATCTACACXXXXXXTCTTTCCCTACACGACGCTCTTCCGATC	

### RNAseq processing and analyses

Total RNA from cells was extracted by the RNeasy Mini Kit (Qiagen) according to the manufacturer’s instructions.

H&E-stained slides of all FFPE blocks were assessed for tumor cell content, and the most suitable FFPE block was selected for DNA extraction. For each patient, six tissue shavings per FFPE block, cut at a thickness of 10 μm each, were submitted for RNA extraction. In cases in which the tumor purity in the selected FFPE block was lower than 70%, vital tumor was enriched by macrodissection, scratching unstained slides after the selection of cellular areas by H&E assessment.

Total RNA from FFPE-macrodissected tissues was extracted by the RNeasy FFPE kit (Qiagen) according to the manufacturer’s instructions on a Qiacube machine (Qiagen).

RNA and further cDNA library quantities were measured using Qubit 2.0 Fluorimetric Assay (Thermo Fisher Scientific) while quality and size were measured by High Sensitivity RNA and DNA screen tapes (Agilent Technologies). Sequencing libraries were constructed starting from 50 to 100 ng of total RNA by optimizations of the QuantSeq 3′ mRNA-Seq Library Prep Kit FWD for Illumina (Lexogen GmbH). DNA libraries were equimolarly pooled at groups of 96 samples and sequenced on a NextSeq 500 high-output, single-end, 75 cycles, v2 Kits (Illumina Inc.).

Sequenced reads were quality checked using fastQC for read mapping and transcript quantification; we used Salmon (v0.8.1) [25], and we use hg38 for indexing reference transcripts. Raw counts and transcripts were normalized with TMM using edgeR. All subsequent analyses were conducted using normalized counts. We corrected batch effect within FFPE and fresh sample with SVA [26] using default parameters. For visualization purposes, we used logCPM.

Differential expression analyses were performed with edgeR, setting FDR < 0.05 and logFC > 0.8 to maximize the following analyses (causal network, downstream effect analyses). Gene groups were identified with WGCNA considering among the module-trait relationships (MTRs) those with high correlation with OSE-like tumors.

Survival analysis was performed considering either the 5-year span overall survival (days elapsed from surgery to death) or the 5-year span disease-free survival (days elapsed from surgery to relapse’s diagnosis), and calculated using the lifelines package [27]. *p* value was computed by a standard Log rank test.

We computed the Cox proportional hazard model using the python package lifelines (version: 0.24.4), considering the relevant metadata as categories (for continuous values, we used a threshold based on quantiles).

We used a combination of causal network analysis, downstream effects analysis, upstream regulator analysis, and molecule activity predictor from ingenuity pathway analysis (QIAGEN Inc. *ingenuity pathway analysis* at https://www.qiagenbioinformatics.com/products/ingenuitypathway-analysis) to identify the predicted functional impact of the genes identified through differential expression or correlation network analysis.

To test the robustness of the transcriptomic-based classification, we used a machine learning approach (python 3.6, sklearn 0.21). First, we defined the training set and test set with a 70–30% ratio, then we calculated automatically the best parameters for the specific set of samples and applied a bagging algorithm with patch selection [28] using decision trees as classifier until the convergence was reached.

### External gene expression dataset classification

To classify the two external HGSOC cohorts (TCGA [29, 30], *n* = 386, RNAseq, and Tothill [31, 32], *n* = 157, microarray), we selected the genes identified as coherently differential expressed between FI-like and OSE-like tumors in both cells and FFPE (*n* = 38 genes). Considering only the selected genes and considering separately the two datasets, we used the Louvain clustering algorithm for community detection [23] changing the size of the communities to identify the most stable clusters (python package scanpy 1.4.5.post3, considered resolution range between 0.1 and 1.5, final value for TCGA = 0.3, final value for Tothill = 0.8). We annotated the clusters identified on diffusion maps and selected the most distant clusters as the most robustly stratified samples (TCGA *n* = 206, Tothill *n* = 102). To assign the correct origin, we considered the concordance of the gene expression values in our stratified datasets.

To apply Tothill’s HGSOC molecular classification [31] to the two datasets, we used the R package consensusOV (version 1.8.1).

CIBERSORT analysis was performed in absolute mode using the online tool available at https://cibersort.stanford.edu, disabling quantile normalization and using the standard LM22 signature to deconvolve the frequency in immune populations in the TCGA dataset. One hundred permutations were performed. The immune cell categories were compared between FI-like and OSE-like tumors by multiple *T* test with FDR correction.

### IHC staining

For immunohistochemical evaluation of T-helper and T cytotoxic lymphocyte prevalence in tumor samples, formalin-fixed, paraffin-embedded specimens were cut into 3-μm sections. Heat-induced epitope retrieval was performed in PTLink Dako with an EDTA buffer (pH 9.0) for 15 min. IHC staining for CD4+ and CD8+ tumor-infiltrating lymphocytes was performed using the anti-human CD4 monoclonal mouse antibody (clone 4B12; dilution 1:300; Dako Denmark A/S, Glostrup, Denmark) and CD8 monoclonal mouse antibody (clone C8/144B; dilution 1:20; Dako). Both immunostains were performed using the EnVision FLEX+ detection system on Autostainer Link 48 (Dako).

Quantification of CD4+ and CD8+ tumor-infiltrating lymphocyte was performed on full-face sections by an experienced pathologist, manually counting immunoreactive cells in 10 high power fields (× 200 magnification). In particular, CD8+ and CD4+ lymphocytes were assessed both in the stromal compartment (including connective tissues surrounding tumor nests, fibro-vascular cores of tumor papillae, and the stroma at the invasive edge of the tumor mass) and in the intraepithelial compartment (lymphocytes in direct contact with epithelial cancer cells) and were recorded separately. Lymphocytes present within tumor necrosis areas were excluded from the assessment.

Since CD4 immunoreactivity is shared by different immune cell species (i.e., T-helper lymphocytes, monocytes, macrophages, and dendritic cells), only CD4-positive cells showing morphological characteristics attributable to lymphocytes were recorded, while all CD8-positive cells were included in the analysis. For each case, the average number of stromal and intraepithelial CD8+ and CD4+ lymphocytes per high power field was provided. For each sample, we derived the average number of cells positive for the considered marker across 10 fields.

For the subgrouping in CD8 high and low, we derived the distribution of the number CD8-positive cells independently of the condition and classified samples according to values higher (CD8 high) or lower (CD8 low) than the median.

PAX8 expression was evaluated on Tissue Micro Arrays (TMAs). For each case, two representative 1-mm spots were selected, and 3 TMAs were designed and built with a semi-automatized Tissue Microarrayer (Galileo TMA CK 3500, Olympus). TMAs were cut into 3-μm sections and stained with the anti-human PAX8 monoclonal mouse antibody (clone BC12, Biocare Medical, Pacheco, CA, USA; dilution 1:100) as described above for anti-CD4 and CD8. Cancer cell nuclear staining intensity was recorded and classified as absent, weak to moderate, and strong.

### Luminex assay

For the detection of chemokines/cytokines, custom Luminex kits (R&D) were used (Table 2). Cells were plated at subconfluency at day 0, changing medium at day 1 and harvesting the supernatant at day 3. Each sample was profiled in duplicate using 50 μl of supernatant per replicate according to the manufacturer’s instructions.

**Table 2:** Cytokines/chemokines profiled in HGSOC samples

List of tested molecules			
CCL2/MCP-1	CCL3/MIP-1 alpha	CCL4/MIP-1 beta	CXCL9/MIG
CCL1/eotaxin	CCL13/MCP-4	CCL17/TARC	CXCL10/IP-10
CCL20/MIP-3 alpha	CCL22/MDC	CCL26/eotaxin-3	CXCL11/ITAC-1
CD25/IL-2 R	CX3CL1/Fractalkine	CXCL1/GRO alpha	CXCL13/BLC/BCA-1
CXCL2/GRO beta	CXCL4/PF4	CXCL6/GCP-2	EGF
GM-CSF	HGF	IFN-gamma	G-CSF
IL-1 beta/IL-1F2	IL-1ra/IL-1F3	IL-2	IL-12 p70
IL-4	IL-5	IL-6	IL-13
IL-7	IL-8/CXCL8	IL-10	IL-15
IL-17/IL-17A	IL-21	IL-23	PDGF-BB
TNF-alpha	TRAIL	CCL5/RANTES	CCL8/MCP-2
FGF basic/FGF-2	IL-1 alpha/IL-1F1	IL-17E/IL-25	VEGF-A
TGF-B1	TGF-B2	TGF-B3	

### RT qPCR

One microgram of RNA from each sample was reverse transcribed into cDNA using the Superscript VILO kit (Thermo Fisher) according to the manufacturer’s instructions. Two hundred nanograms of cDNA was analyzed by Taqman (Thermo Fisher) qPCR probing for DDR2 (Hs01025957), PDGFRA (Hs00998018), SEMA6D (Hs00227965), SLITRK4 (Hs00331273), MAL2 (Hs01043579), PAX8 (Hs01015257), PMEPA1 (Hs00375306), TNS4 (Hs00262662), SCNN1A (Hs00168906), and GAPDH and ACTB (Hs02758991 and Hs0160665, normalizers) using the SsoAdvanced master mix (Biorad). qPCR was run on a LightCycler 480 (Roche) using the standard amplification protocol for 45 cycles, and the average of the Ct of GAPDH and ACTB was used as normalizer. Six independent samples were analyzed in technical triplicates.

## Results

**Fig. 1:**
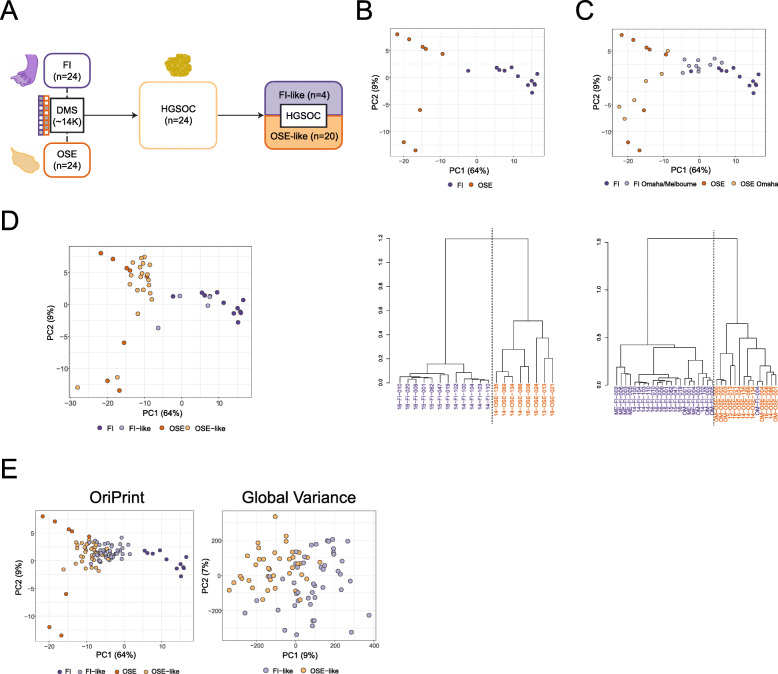
OriPrint is able to stratify HGSOC on the basis of its cell of origin. **a** Schematic representation of the experimental pipeline. FI, fimbrial epithelium; OSE, ovarian surface epithelium; HGSOC high-grade serous ovarian cancer; FI-like, tumors originating from the fimbrial epithelium; OSE-like, tumors originating from the ovarian surface epithelium. **b** Top: PCA analysis of FI and OSE samples (*n* = 11 and 8, respectively) from the IEO cohort (purple and orange, respectively) considering ORIPrint CpGs. Bottom: Hierarchical clustering of the same samples, distance = Pearson’s correlation. **c** Top: PCA analysis of FI and OSE samples (tones of purple and orange, respectively) from IEO, Omaha (*n* = 5 and 5, respectively) and Melbourne (*n* = 6 FI) cohorts considering OriPrint CpGs in the space defined by normal samples. Bottom: Hierarchical clustering of the same samples, distance = Pearson’s correlation. **d** PCA analysis of normal and tumor samples (*n* = 24) from the IEO cohort, annotated using Pearson’s correlation-based classification in the space defined by normal samples. **e** PCA analysis of normal samples from IEO cohort and tumor samples from the Melbourne cohort (*n* = 85), annotated using Pearson’s correlation-based classification both in the space defined by OriPrint and normal samples (left) and by the whole set of CpGs (right)

In order to probe whether DNA methylation could be used as a developmental tracker for HGSOC’s cell of origin, we derived genome-wide DNA methylation profiles of short-term cultures of the fimbrial epithelium (FI, *n* = 12), ovarian surface epithelium (OSE, *n* = 8), solid tumor-derived (HGSOC, *n* = 11), and ascites-derived (AS, *n* = 13) tumor cells. We checked in our dataset as well as in two independent published datasets generated from frozen tissues, the Omaha [7] and Melbourne [16] cohorts, whether the global variance in DNA methylation could already stratify tumors on the basis of their cell of origin. To this end, we used Uniform Manifold Approximation and Projection (UMAP) visualization, a non-linear dimensionality reduction technique that preserves the global structure of the data [33], to capture the largest fraction of variability in DNA methylation. We found that neither in published datasets nor in our cohort was it possible to bipartition tumor samples according to FI or OSE global methylation (Additional File 1: Fig. S1), since most of the variability is driven by the differences existing between normal and tumor samples. We thus reasoned that, given such a distribution of variance, we could enhance the potential sensitivity of DNA methylation as a cell-of-origin tracker by first defining the specific subset of differentially methylated CpGs able to distinguish FI and OSE. We thus identified 13,926 differentially methylated sites (DMS) between the two normal cell types in culture (Fig. 1a) (henceforth OriPrint), composed of 8613 hypermethylated CpGs and 5313 hypomethylated CpGs in OSE, and which were enriched for open sea CpG areas [34] and were functionally annotated mostly to introns and intergenic regions (Additional File 1: Fig. S2). Next, we determined whether this blueprint, which was derived from cells in culture, was able to segregate correctly FI and OSE tissues (Fig. 1b). As shown in Fig. 1c, a hierarchical clustering based on Pearson’s correlation confirmed that the tissue samples were correctly divided into FI and OSE, thus proving that our culture conditions recapitulated the complexity and preserved the essential epigenetic properties of tissues in vivo. Indeed, the very same FI sample form the Omaha cohort that has already been misclassified as OSE in the original published dataset [7] behaved in the same manner also in our classification (Fig. 1c), underscoring the robustness and sensitivity of our classifier.

To see whether OriPrint could be used to stratify HGSOC samples, we applied the same methodology to our cohort of tumor samples and showed that tumors were bipartitioned through the OriPrint in two classes that we define as FI-like and OSE-like tumors (Fig. 1d, Additional File 1: Fig. S3).

Next, to verify that our approach could be translated to independent tumor cohorts as well as to frozen biopsies, we applied the OriPrint classifier to the Melbourne cohort and analyzed the global variance in DNA methylation across FI-like and OSE-like HGSOC. Interestingly, by principal component analysis, we could show that the superimposition of the categories defined by OriPrint identified two contiguous but distinguishable clusters in the first two components of variance, thus indicating that the cell of origin is the first responsible of the variability existing in DNA methylation between HGSOC samples (Fig. 1e). This distinction was also evident when using alternative visualization methods (Additional File 1: Fig. S4).

**Fig. 2:**
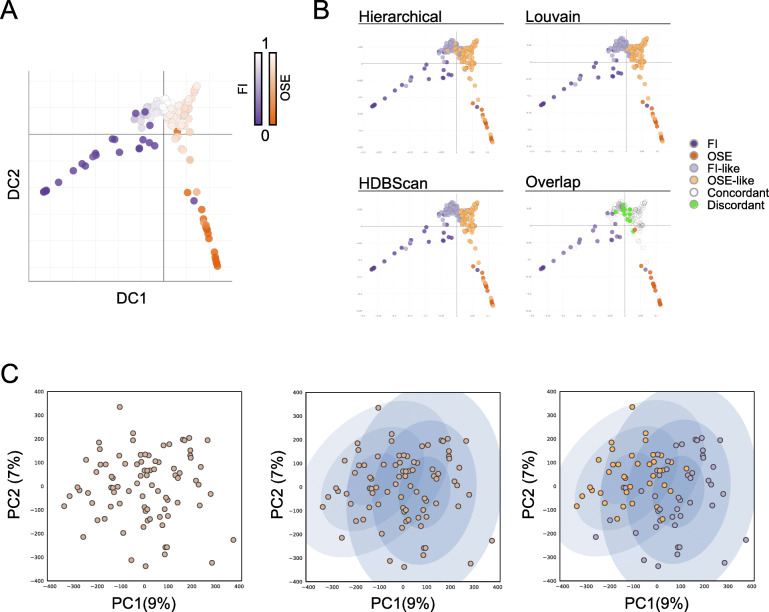
OriPrint is a solid stratifier and establishes the tissue of origin as a major source of variance for HGSOC. **a** Diffusion map with pseudotime timeline performed on OriPrint CpGs for samples of all cohorts. The origin is situated either in the distal FI (purple origin) or OSE (orange origin) samples. **b** Diffusion maps showing the classification output for the three indicated clustering methods. The overlap plot shows in white the samples that are concordantly classified by all three methods and in green the samples that have a different classification in at least one of the three methods. **c** PCA analysis coupled to Gaussian Mixture Model (GMM) clustering of the Melbourne tumor cohort. Left: First two components of global variance in DNA methylation for the considered samples. Middle: The two probability distributions calculated by GMM. Right: Overlay of the OriPrint classification, showing a consistent overlap with GMM’s distributions

Together, these results show that OriPrint is able to robustly stratify HGSOC tumors into FI-like and OSE-like subtypes across independent clinical cohorts and sample processing pipelines. To further confirm this finding, we used diffusion map coupled to pseudotime analysis, which was previously used for single-cell RNA sequencing data to derive the developmental progression of cells and identify branching decisions and differentiation endpoints [35, 36], to highlight whether an evolutionary timeline exists between tumors and the two origins. Using OriPrint and setting the origin in both OSE and FI samples, we calculated the two pseudotime lines and could show that HGSOC samples are a mandatory step in pseudotime evolution between the two normal tissues. Moreover, we showed that the intersection between the two paths occurs centrally in the tumors’ distribution (Fig. 2a), thus further confirming that both origins are plausible for HGSOC. Instead, using the whole set of CpGs, we could not score any clear evolution between FI and OSE and HGSOC (Additional File 1: Fig. S5), thus proving that OriPrint is necessary to stratify tumors according to their cell of origin.

In order to determine whether the classification was solid and not bound to the clustering method used, we applied two additional clustering methods, Louvain [23] and HDBScan [24], to stratify HGSOC and checked the concordance of classification by OriPrint. We could show that, overall, we could achieve > 92% concordance of classification among the three clustering methods used, thus proving the robustness of this approach to define FI and OSE-originated tumors (Fig. 2b).

Next, we sought to determine whether, by this method, the same conclusions could be drawn directly from the full set of CpG or if OriPrint was necessary to define the classification into FI-like and OSE-like tumors. We applied Gaussian Mixture Model (GMM) clustering to the entire set of CpG in the space derived from the first two components of variance (i.e., the components accounting for the highest variance in the system) and were able to derive two probability distributions for samples. We then overlaid the classification derived from OriPrint and could show that the two clustering methods are mostly superimposable (Fig. 2c). Thus, we could show that HGSOC cell of origin is one of the main determinants of the differences in DNA methylation existing among HGSOC samples.

### Stratification of a retrospective HGSOC cohort

**Fig. 3:**
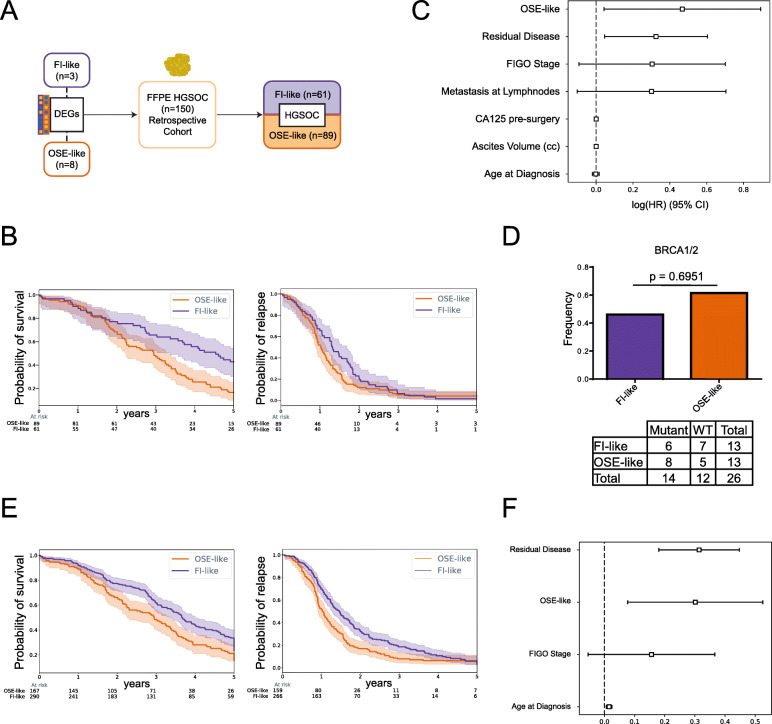
The cell of origin of HGSOC impacts the prognosis of patients independently of *BRCA1/2* mutations. **a** Schematic representation of the strategy based on RNAseq to stratify a retrospective cohort of HGSOC. **b** Overall (left) and disease-free (right) survival of patients stratified by the cell of origin of HGSOC. Light-colored areas represent confidence intervals. Log rank test was used for statistical significance. **c** Cox’s proportional hazard model on clinical data. **d** Mutational status of *BRCA1/2* in the retrospective cohort classified in FI-like (purple) and OSE-like (orange) tumors, shown as mutational frequency (top barplot) and contingency table (bottom). Fisher’s exact test was performed based on the contingency table. **e** Cumulative overall (left) and disease-free (right) survival over 5 years across the IEO, TCGA, and Tothill datasets. **f** Cox’s proportional hazard model on clinical data from the union of IEO, TCGA, and Tothill’s cohorts

In order to check whether the two subtypes had a different impact on patients’ prognosis, we decided to stratify a well-characterized FFPE cohort of HGSOC (*n* = 150 independent patients, Istituto Europeo di Oncologia (IEO) cohort) (Additional File 2: Table S1). Since FFPE fixation compromises bisulfite-based DNA methylation analysis, we concentrated our effort on analyzing transcriptomic profiles that we obtained from our cohort of samples in culture and from macrodissected FFPE tissues. We then used the differentially expressed genes between FI-like and OSE-like tumors, previously assigned to either of the two tissue of origin through DNA methylation (*n* = 3 FI-like, *n* = 8 OSE-like), to stratify the cohort and to perform survival analysis (Fig. 3a). Using this method, we found that the patients affected by OSE-like tumors had a worse prognosis, as assessed by overall survival analysis (difference at median survival 1.6 years, *p* value < 0.0007). Instead, we could not score an impact on the disease-free survival of patients (difference at median recurrence 0.3 years, *p* value < 0.07) (Fig. 3b), indicating that both subtypes are equally prone to recurrence in vivo. Additionally, we performed Cox multivariate analysis, showing that the tissue of origin of HGSOC is the main determinant of patients’ survival (Fig. 3c, Additional File 2: Table S2).

To check whether the difference in overall survival was due to the presence of subtype-specific mutations, we analyzed the mutational status of *BRCA1* and *BRCA2* (*BRCA1/2*), which has been shown to positively correlate with patient’s survival [29], in a subset of samples from patients who underwent genetic testing as part of their clinical evaluation and family history. We could show that *BRCA1/2* mutations are present in both subtypes with no difference in frequency in the two groups, thus indicating that the mutational status of these genes is not responsible for the difference in survival existing between the two groups (Fig. 3d).

To test the robustness of the RNA-based stratification method, we used a machine learning approach [28, 37] aimed at defining the samples that are consistently classified as FI-like and OSE-like. More in detail, we permuted 1000 times the genes used for the generation of the model that was then applied to the test set to derive the classification into our categories. We then shuffled 1000 times the samples to be used for the training set and test set and reiterated the stratification method. The samples that cumulatively did not reach 75% classification consistency across iterations were classified as “uncertain” (*n* = 22, 14.5%). We then compared the overall survival curves of the newly defined FI-like and OSE-like tumors (88% consistent with the previous stratification method) and could confirm a significantly worse prognosis in patients affected by OSE-like tumors (Additional File 1: Fig. S6A), thus showing the robustness of our approach.

Next, we used transcriptomic data to apply our stratification approach to two published datasets, the TCGA cohort [29] and the Tothill cohort [31]. Specifically, the TCGA cohort entails FFPE samples profiled at the transcriptomic (RNAseq) and DNA methylation level but limited to the 27k array platform resolution, which does not provide sufficient coverage for the application of OriPrint. Tothill’s cohort, instead, provides only microarray expression data. Thus, in order to define the most universal and robust transcriptional signature to be translated across sample preparation (i.e., whole slice FFPE versus macrodissected specimens) and transcriptomic profiling methods (RNAseq versus microarrays), we derived a new signature defined by genes that were differentially expressed between FI-like and OSE-like tumors, in both cells and FFPE samples. We then applied the Louvain algorithm to cluster our samples and used diffusion maps to reveal the most separated subsets of samples in both cohorts (TCGA and Tothill) and thereby identify FI-like and OSE-like tumors (TCGA cohort: FI-like 172, OSE-like 34, uncertain 180; Tothill’s cohort: FI-like 58, OSE-like 44, uncertain 55). Next, we performed survival analysis independently on both datasets and found that patients affected by OSE-like tumors show a consistently worse trend in overall and disease-free survival (Additional File 1: Fig. S6B-C, Additional File 2: Table S3). In addition to this, by multivariate analysis, we found that the main risk factor for adverse prognosis in the TCGA cohort is OSE-derived HGSOC (Additional File 1: Fig. S6D-E, Additional File 2: Table S2), thus corroborating our findings from the IEO cohort. Also, by performing overall and disease-free survival analysis on the union of the three datasets, we found a statistically significant difference in both parameters over the course of the entire follow-up (overall survival: difference at median 8.5 months, *p* value = 0.0006; disease-free survival: difference at median 5.2 months, *p* value = 0.0002) (Fig. 3e, Additional File 2: Table S3). Finally, we performed multivariate analysis using the common factors available for the three datasets and could confirm OSE-derived HGSOC as one of the main risk factors for HGSOC patients (Fig. 3F and Additional File 2: Table S2). Taken together, these findings confirm the tissue of origin as a main determinant for patient’s prognosis.

Finally, we checked in the TCGA cohort whether there were differences in terms of mutational landscape between the two tumor subtypes, but could not find any statistically different frequency in mutated genes (Additional File 2: Table S4). Interestingly, we found that the two tumor subtypes carried differential copy number burden (fraction of the genome altered), with OSE-like tumors being less impacted by copy number gain/loss (Additional File 1: Fig. S7). This result is possibly indicative of a less impacted DNA damage response in OSE-like tumors resulting in a worse response to carboplatin/paclitaxel treatment [38].

### Definition of subtype-specific transcriptional signatures

To gain insight into the specific transcriptional features that characterize the most detrimental HGSOC, i.e., OSE-like tumors, we performed Weighted Gene Correlation Network Analysis (WGCNA) on all sample categories (FI, 6: FI-like, 3; OSE, 8; OSE-like, 8 samples) and identified 1378 genes whose levels of expression correlate specifically in OSE-like tumors.

**Fig. 4:**
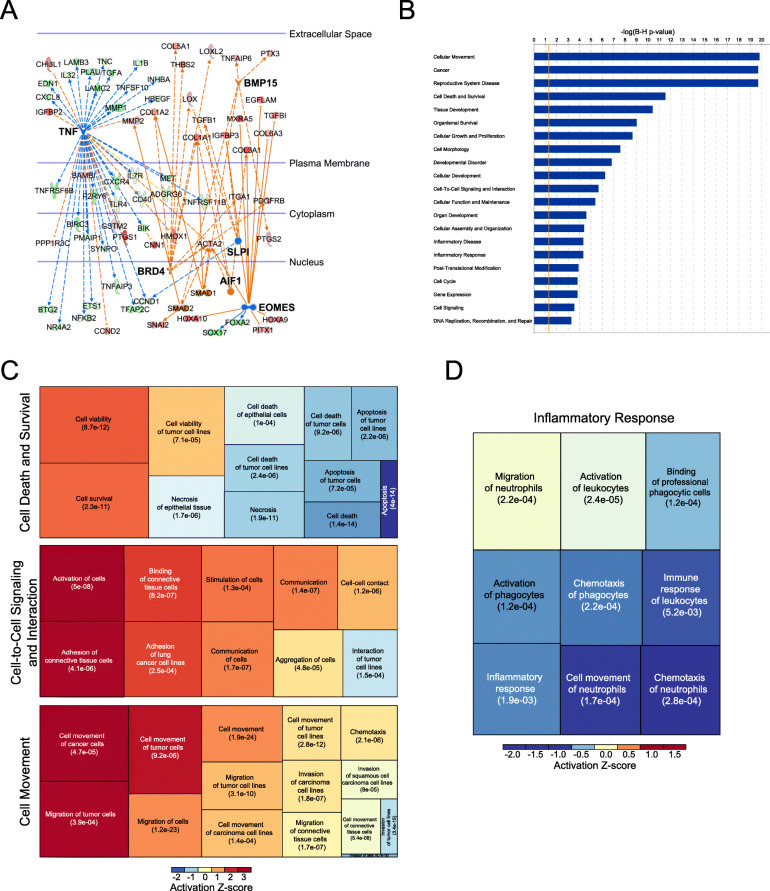
Gene expression patterns of OSE-like tumors reveal a lower inflammatory response coupled to increased survivability and active cell-to-cell signaling. **a** IPA causal network analysis performed on WGCNA eigengenes associated with OSE-like tumors. Blue: regulator genes whose pathway is predicted to be inhibited; orange: regulator genes whose pathway is predicted to be activated; red: upregulated eigengenes; green: downregulated eigengenes. **b** IPA disease and function enrichment analysis on WGCNA eigengenes associated with OSE-like tumors. Enrichment *p* values are shown after Benjamini-Hochberg FDR correction. **c** Treemap of three of the categories in **b**. Box dimension is derived on activation *z-*score. Enrichment *p* values are shown as in **b**. **d** The inflammatory response IPA disease and function enrichment analysis category predicted to be inhibited in OSE-like vs FI-like tumors

In order to identify the upstream regulator pathways that could account for the specific gene expression pattern of these tumors, we performed IPA causal network analysis on WGCNA genes. Through this approach, we identified 6 pathways that were regulating 80 genes specific for OSE-like tumors (TNF, BRD4, EOMES, BMP15, AIF1, SLPI). Specifically, the TNF pathway is predicted to be deactivated in OSE-like HGSOC, suggesting a reduced inflammatory response elicited by these tumors, while the other pathways are predicted to be activated and associated to extracellular matrix remodeling and WNT/β-catenin activity (Fig. 4a).

To understand the effect of the genes identified by WGCNA on OSE-like tumors’ fitness, we performed IPA disease and function analysis. This highlighted a consistent inactivation of the cell death-related categories, with a concomitant activation of the cell survival-related genes, and the activation of categories related to cell-to-cell signaling and interaction and cell movement (Fig. 4b, c). Taken together, these data suggest higher fitness and survival for OSE-like tumors.

Thus, in order to probe the consequences of the pathways’ alterations identified in FI-like and OSE-like tumors, we proceeded to a differential expression analysis between these two subtypes. This uncovered 146 differentially expressed genes (72 downregulated genes and 74 upregulated genes in OSE-like tumors) that distinguished the two categories, consistently associated with decreased inflammatory response in OSE-like tumors, thus confirming our observations in WGCNA (Fig. 4d).

**Fig. 5:**
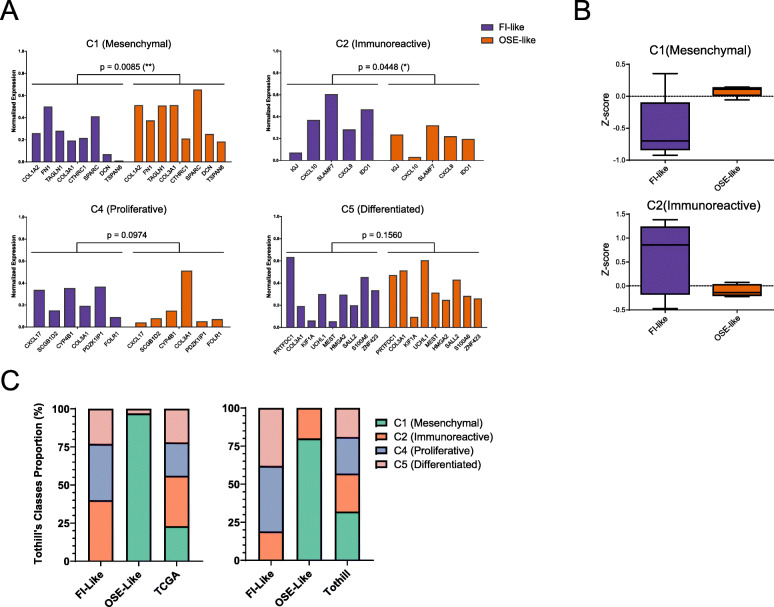
OSE-like tumors have a mesenchymal, non-immunoreactive molecular phenotype. **a** Barplot of the mean expression levels of HGSOC molecular signatures in FI-like (purple bars) and OSE-like (orange bars) tumors. Two-way ANOVA analysis was used to calculate the significance for the difference in expression of the signature in the two groups. **b** Boxplot of the *z*-score relative to the expression of the mesenchymal (top) and immunoreactive (bottom) signatures in FI-like (purple) and OSE-like (orange) tumors. **c** Frequency stacked barplot for the proportion of Tothill’s molecular classes in FI-like and OSE-like tumors considering TCGA cohort (left panel) and Tothill’s cohort (right panel). The distribution in the entire considered dataset is reported on the rightmost bar of each panel (TCGA and Tothill, respectively)

To further dissect the molecular features that characterize these two tumor subtypes, we took advantage of the published molecular classification for HGSOC by Tothill and colleagues [31] that identified four molecular subclasses (C1—mesenchymal, C2—immunoreactive, C4—proliferative, C5—differentiated). We used the minimal signature of validated classifier genes [39] that were expressed in our dataset and found that OSE-like tumors resembled mesenchymal tumors, while skewing away from the immunoreactive phenotype (Fig. 5a, b). Moreover, we checked whether also the stratified TCGA and Tothill’s cohort showed an enrichment for any of the molecular subclasses and found that, consistently with our cohort, OSE-like tumors are strongly enriched in the C1 mesenchymal category (*p* = 1.89e−26 and 4.24e−15 hypergeometric test, respectively) (Fig. 5c), thus further corroborating our findings.

### OSE-like tumors have an immunomodulatory phenotype

**Fig. 6:**
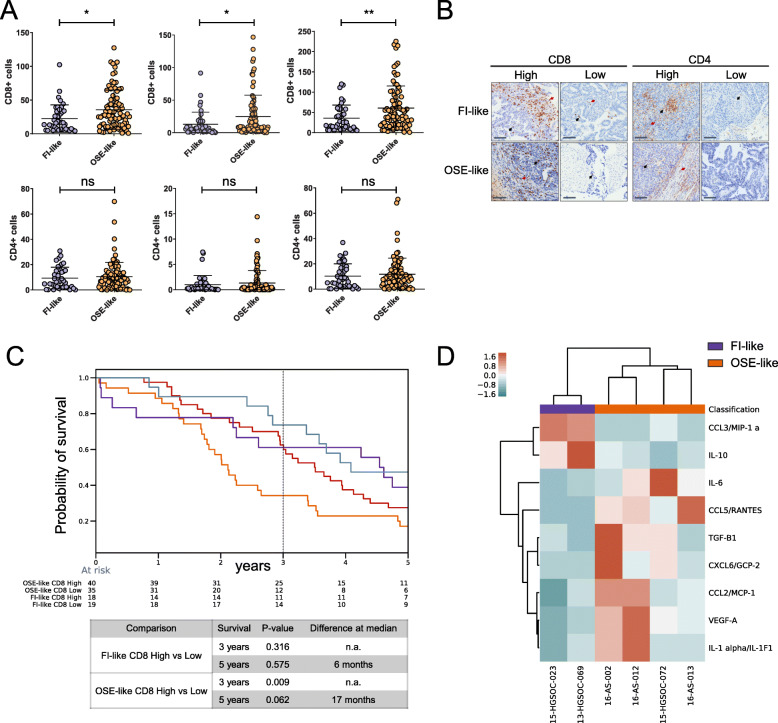
OSE-like tumors show an immunomodulatory phenotype. **a** Dotplot showing the number of CD8+ cells (top panels) and CD4+ cells (bottom panels) in stromal and intratumoral regions. Mann-Whitney *U* test was used to derive statistical significance (*p < 0.05, **p < 0.01, ns = non-significant). **b** Immunohistochemistry staining for CD8 and CD4 in FI-like and OSE-like tumors. Representative pictures are shown. Red arrows: stromal positive cells. Black arrows: intratumoral positive cells. Scale bar = 100 μm. **c** Kaplan-Meier overall survival curves for FI-like and OSE-like affected patients, subdivided in CD8 high and low. The dashed line is set at 3-year survival. Log rank test results for significance are shown in the bottom table. **d** Heatmap of the level of protein expression of cytokines/chemokines segregating FI-like (purple) and OSE-like (orange) tumors. Distance = Euclidean

In order to further investigate the OSE-like immunomodulatory transcriptional phenotype (Figs. 4 and 5) in relation to its worse prognosis, we first performed IHC staining for cytotoxic (CD8+) and T-helper (CD4+) lymphocytes for the full cohort of FFPE samples. We observed an increased amount of CD8+ cells in OSE-like tumors as compared to FI-like tumors in both the stromal and intratumor compartment, while we could not detect any difference in the CD4+ population amounts among the two groups (Fig. 6a, b). As the tumor-immune crosstalk for HGSOC is still largely uncharted, these results prompted us first to understand whether a higher amount of cytotoxic T lymphocytes could impact patients’ prognosis, subdividing FI-like and OSE-like groups into CD8+ high and low and performing overall survival analysis. We did not score any difference in survival at 5 years, while at 3 years, we found that higher amounts of CD8+ cells in OSE-like tumors correlated with an initial positive impact on patients’ prognosis (Fig. 6c). Hence, while OSE-like tumors are able to attract CD8+ cells, the extent of their recruitment does not account for the patients’ overall worse prognosis, despite suggesting that OSE-like tumors shape their immune microenvironment.

To further investigate a possible re-wiring of the immune compartment by OSE-like tumors towards immune suppression, we applied the CIBERSORT algorithm [40] to TCGA samples to deconvolute the immune composition of HGSOC. By comparing FI-like and OSE-like tumors, we found increased amounts of memory resting T cells and M2 polarized macrophages in OSE-like tumors (Additional File 1: Fig. S8), the latter previously associated to immune suppression in several types of cancers [41].

Finally, to understand whether OSE-like and FI-like tumors were also characterized by differential immune signaling, we performed Luminex assay screening for 51 cytokines/chemokines in the supernatant of cultured cells. Among those, we were able to identify 9 proteins that were able to distinguish between the two groups (Fig. 6d). While CCL3 and IL10 (upregulated in FI-like tumors) have been described as molecules able to elicit antitumor response [42, 43], the molecules upregulated in OSE-like tumors have all been associated with the induction of immunosuppressive response and increased tumor invasiveness [44–48]. These results confirm that OSE-like tumors have an immune secretory landscape that is compatible with the suppression of immune response and with a negative impact on patients’ prognosis.

### PAX8 is differentially regulated according to the origin of HGSOC

Last, we probed the correlation between gene expression and DNA methylation across FI-like and OSE-like to test whether, beyond its cell-of-origin-based classifying function, the epigenetic traces from OriPrint could also reveal further biological insight about the two tumor types. We found 38 genes that were both differentially expressed and methylated at the promoter level between FI-like and OSE-like HGSOC and validated their expression by qPCR (Additional File 1: Fig. S9A-B). Among them we focused on the paradigmatic case of PAX8, given its historical record as a defining marker for HGSOC [7, 49, 50] whose expression is shared with the fimbrial epithelium. Indeed, this latter feature has been proposed as one of the demonstrations of the Müllerian origin of HGSOC. However, precisely since not all HGSOC samples express PAX8, we sought to determine whether PAX8 expression could be related to the cell of origin of this disease.

**Fig. 7:**
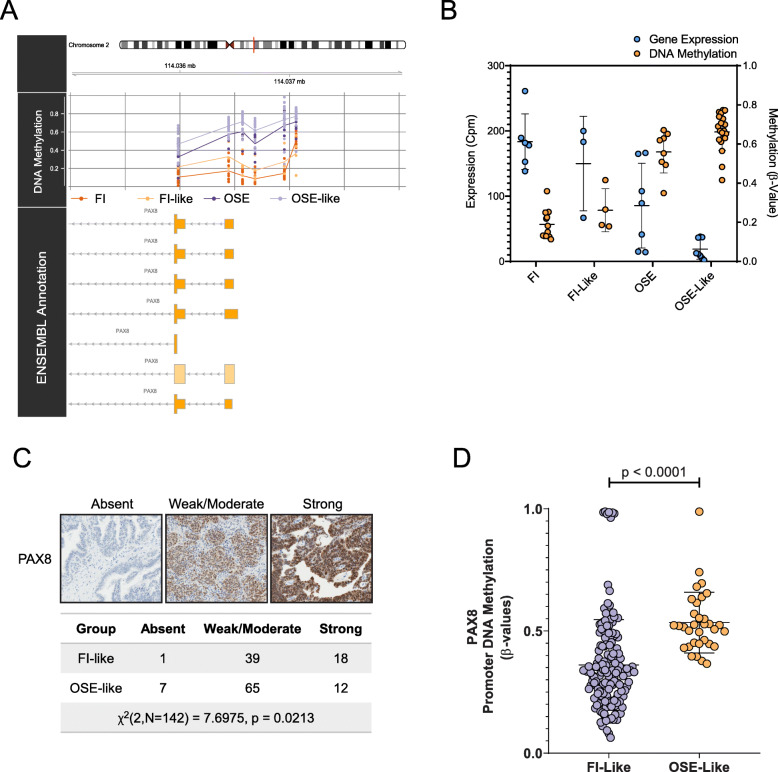
PAX8, a defining marker of HGSOC, is differentially methylated and expressed in FI-like vs. OSE-like tumors. **a** Graphical representation of the methylation of CpGs in PAX8 promoter across the indicated sample groups**. b** Dotplot depicting the gene expression level of PAX8 by RNAseq (blue bars, left *Y*-axis) and the DNA methylation level of its promoter by array (orange bars, right *Y*-axis) in the considered categories. The table summarizes the results of Mann-Whitney *U* tests (two-tailed) performed in the indicated comparisons. Data are shown as mean ± standard deviation. **c** PAX8 IHC performed on a tissue microarray of FFPE HGSOC. Samples were divided according to staining intensity. FI-like and OSE-like tumors were compared for the enrichment in the indicated categories by chi-square testing. **d** Dotplot depicting the DNA methylation level of PAX8 promoter in TCGA samples stratified as FI-like and OSE-like tumors. Mann-Whitney *U* test (two-tailed) for significance

We indeed found that PAX8 is expressed in FI but not in OSE samples and remains differentially expressed between FI-like and OSE-like tumors, thus recapitulating the expression pattern from the tissues of origin. To understand whether this pattern of expression was reflected also at the regulatory level, we analyzed the level of methylation of its promoter in our samples. Consistent with the differences in gene expression, PAX8 was differentially methylated among FI and OSE samples and, also, between FI-like and OSE-like tumors, in an anticorrelative fashion with gene expression (*r* = − 0.85) (Fig. 7a, b). We validated these findings through qPCR and targeted bisulfite sequencing (Additional File 1: Fig. S9C-D), thus confirming a differential regulation for this gene aligned to the cell of origin of this disease. To verify that this difference was true also at the protein level, we performed immunohistochemistry analysis on a tissue microarray encompassing 142 samples from our cohort of FFPE samples. As expected, we found that FI-like tumors were enriched in tumors expressing high levels of PAX8 (Fig. 7c) when compared to OSE-like tumors.

Finally, we checked also in the TCGA cohort the levels of DNA methylation of the 38 differentially expressed/methylated genes (Additional File 1: Fig. S10) originally identified in our in-house cohort by OriPrint. Only 19 of them were covered in the TCGA 27k array and showed the same pattern of differential methylation between FI-like and OSE-like, a difference that reached statistical significance for 9 of them. Of note, PAX8 was differentially methylated between FI-like and OSE-like tumors also in this setting, showing a consistent difference between the two subtypes (Fig. 7d) that confirms PAX8 as an origin-specific marker for FI-like tumors.

## Discussion

High-grade serous ovarian cancer (HGSOC) is a major unmet need in oncology. The lack of suitable human experimental systems that recapitulate the pathogenesis of the tumor is a well-recognized cause of the negligible progress over the last decades [3, 4], with virtually no improvement in patients’ outcome since the introduction of cis-platin as first-line treatment in the 1980s [3, 4]. In this regard, the persistent uncertainty regarding the tissue of origin represents a particularly conspicuous hurdle for the elucidation of the molecular pathogenesis as a rational basis for the development of targeted therapies.

In contrast to the previously widely accepted “incessant ovulation” hypothesis [51], according to which tumors could arise in ovaries as the result of the continuous break/repair of the ovarian surface epithelium, more recent evidence had been increasingly emphasizing the role of the distal tract of the fallopian tube as the most likely tissue of origin of HGSOC, largely as a result of the identification of STIC carrying a core subset of mutations present in both primary and metastatic tumors from the same patient [8]. To this respect also, the incessant ovulation hypothesis has been revisited, with emphasis on the possible role of the follicular fluid in creating a pro-inflammatory environment in the tuba during oocyte capture [51]. Additionally, the continuous break/repair cycle of the ovarian surface epithelium could favor the implant of tumor cells shedding from the fimbria, which in turn could further develop inside the ovary as primary inclusion cysts and, in a second moment, as full-fledged HGSOC. Concomitantly, however, phylogenetic mutational analysis from different studies posited that a significant fraction of STIC (25%) could actually represent metastatic rather than primary lesions [9]. Along with clear evidence from murine models showing that genetic alterations in both fallopian tube and ovarian surface epithelia can drive tumorigenesis, this contributed to an enduring uncertainty about the relative oncogenic contribution of either epithelium, a situation unlike that of virtually any other solid tumor thanks to the molecular progress of the last decade [52–58]. Here we tackled and solved this problem by analyzing DNA methylation, given recent evidence pointing to its conservation across normal tissues and their corresponding tumors that warranted its use as a developmental stratifier in multiple contexts [12, 13]. We thus derived DNA methylation profiles for both normal (FI and OSE) and tumor (HGSOC and AS) samples. While a global DNA methylation analysis, as in approaches adopted by previous more limited studies [7], was not able to segregate tumors in a tissue of origin-consistent manner (capturing instead solely the differences between normal and tumor samples), we demonstrate that a strategy based on the prior identification of differentially methylated sites between FI and OSE (OriPrint) allows the robust bipartition of HGSOC, in both cultured cells and whole frozen tissues. Moreover, the identified categories reflect the global variability of HGSOC, thus assigning for the first time a role for the cell of origin in determining the heterogeneity among different tumor samples.

The a priori epigenetic stratification in FI-like and OSE-like HGSOC allowed us to interrogate a larger retrospective cohort to define differentially expressed genes between these two categories. Specifically, to interrogate the clinical relevance of our stratification, we resorted to RNAseq profiling of a retrospective cohort of macrodissected FFPE HGSOC samples, employing a shallow 3′-UTR RNAseq approach that allowed us to reduce the library preparation and sequencing costs down to the range of 100 € per sample, thus demonstrating the feasibility of such a stratification for clinical translation. In particular, this approach allows the cost-effective transcriptomic characterization of tumor subtypes through the detection of ~ 15,000 genes, thus avoiding the reduced sensitivity/specificity bound to the restriction to minimal signatures usually employed for classification [59]. By this approach, we found that OSE-like tumors have a negative impact on patients’ prognosis, a finding confirmed also on the TCGA and Tothill’s cohorts and consistent with previous reports [57, 58]. While the differences in survival between patients affected by FI-like and OSE-like HGSOC are mostly not statistically significant when considering the datasets separately, we can attribute this to the more heterogeneous composition of HGSOC samples in these two cohorts (i.e., whole slice FFPE vs. macrodissected tissues) and to the alternative transcriptomic profiling platform used in Tothill’s cohort (i.e., gene expression microarrays). Moreover, our gene expression signature has been applied without performing any batch correction among datasets, further reinforcing the universality of our strategy. Future studies aimed at the identification of common features existing between different sample types and profiling approaches based on deep learning should allow the integration of several datasets to better investigate the effect of the tissue of origin on patients’ prognosis. Interestingly, we also found that there is a specific enrichment/skew, in FI-like versus OSE-like tumors, of previously described molecular subtypes of HGSOC [31]. In particular, we identified the C1 mesenchymal category, which has been previously described as encompassing more aggressive cases with poor outcome, as being the main subtype within OSE-like tumors. This evidence is in line with the survival status of patients in our cohort, with similar trends in TCGA and Tothill’s cohort, and with previous reports on integrated publicly available microarray data, linking the OSE origin to a more mesenchymal phenotype [57]. In contrast with this, two other studies previously linked the mesenchymal category to FI-derived tumors [55, 56]. The most parsimonious understanding of this discrepancy can be attributed to the fact that these studies were neither aimed at the generation of a univocal classifier (i.e., a statistical or computational method to assign from a probability score exclusively either one or the other origin to samples) nor at evaluating its prognostic significance, being rather based on a scoring system determining the similarity of each sample to either the two tissues of origin. Also, while our strategy is based on the differential expressed genes between previously DNA methylation-stratified FI-like and OSE-like tumors, thus directly reflecting the specific expression features of the two tumor subtypes, these studies were heavily relying on genes differentially expressed between normal tissues. The different stratification outcome could be thus reflecting the overriding differences existing between the normal tissues rather than between the two tumor subtypes. Regardless, consistent with previous findings, our stratification strategy confirmed the heavy shift in ratio between FI- and OSE-like tumors in published datasets towards FI-derived tumors. This is contrasting with the higher representation of OSE-like tumors in the IEO cohort and highlights a cohort-dependent incidence of tumors derived from each origin. Extended studies, aiming at the stratification of tumors in datasets derived from different geographical areas, will shed light on the possible epidemiological features that result in such diverse incidence of these two tumor subtypes.

*BRCA1/2* mutations have previously been linked to increased survival of patients with HGSOC [29]. To check whether the increased survival of FI-like HGSOC affected patients could be due to the co-occurrence of *BRCA1/2* mutations, we analyzed the occurrence of these mutations on a subset of either tumor subtype, which were derived from patients who underwent genetic testing as part of their clinical evaluation and family history. Despite the higher rate of *BRCA1/2* mutation in this subset, which is likely due to the specific subset we considered, we found that *BRCA1/2* mutations occur in both types of tumors, thus excluding that these genes are mutated exclusively in the FI-like subtype. Another important aspect linked to the DNA damage response (DDR) is the emerging concept of “BRCAness,” according to which tumors can be characterized by mutations/gene inactivation/gene expression patterns whose outcome is similar and closely related to the mutational status of *BRCA1/2* [60]. While we cannot exclude that our phenotype segregates with the BRCAness phenotype in its full extent, the comparable distribution of *BRCA1/2* mutations among FI-like and OSE-like HGSOC should orient future studies towards the dissection of the role that other alterations in the DDR pathway may potentially contribute to the difference in survival between patients affected by two tumor subtypes that we identified. To further investigate potential differences in the mutational landscape of FI-like and OSE-like tumors, we checked in the TCGA cohort whether any difference could be found in the genetic profiles of these two tumor subtypes. Compatible with a common developmental ancestor, whose influence on the epigenetic landscape could result in a similar permissiveness to genetic insult, we found that there was no statistically significant difference in terms of frequency of mutations. Interestingly, we found a differential copy number burden between FI-like and OSE-like tumors, the former presenting higher FGA. This could be further corroborating the hypothesis that DDR could be more impacted in FI-like tumors, thus increasing genomic instability and conferring better response to platinum therapy in patients affected by this tumor subtype. Indeed, recent in vivo studies are showing better response to carboplatin/paclitaxel treatment for FI-derived tumors [55, 61]. This is consistent with the better prognosis of patients affected by FI-like tumors in the IEO cohort, the latter being characterized by patients treated homogeneously with standard carboplatin/paclitaxel regimens.

In order to gain deeper insight into the molecular features of OSE-like tumors, we analyzed both WGCNA genes and genes that were differentially expressed between FI-like and OSE-like tumors. We identified reduced inflammatory response, higher cell viability, increased cell-to-cell signaling, and motility as paradigmatic features of OSE-like tumors. In particular, this is supported by recent evidence showing that the amount of tumor-infiltrating lymphocytes (TILs) directly correlates with patients’ prognosis [62]. Moreover, the reduced cell death coupled to increased viability and higher cell movement are fully compatible with a more aggressive phenotype in these tumors. We validated these results by cytokine/chemokine profiling, T lymphocyte staining, and CIBERSORT analysis, showing that OSE-like tumors present, indeed, an immune-modulatory phenotype. Specifically, our analysis shows that CD8+ lymphocytes are recruited to the tumor site and this mitigates the worse prognosis OSE-like tumors in the short term, consistently with the published literature [62]. At the 5 years’ benchmark, the worse prognosis of OSE-like tumors remains however unaffected by the extent of CD8+ infiltrate, pointing to the possibility that active immunosuppression could eventually set in and contribute to the worse outcome of this specific tumor type. This is consistent also with our inference, in the TCGA dataset, of increased recruitment of M2 macrophages that are well-known repressors of the host’s immune response against the tumor [41] and with the expression of immunosuppressive cytokines that we scored from OSE-like samples. Taken together, these results can now pave the way to a systematic, tissue-of-origin tailored analysis of the HGSOC-immune system crosstalk, hopefully informing the rational application of immune-checkpoint therapies [63, 64] in the context of our refined stratification of HGSOC patients.

Finally, among differentially expressed genes between FI-like and OSE-like tumors, we identified 38 genes that are also differentially methylated at the promoter level, in an anticorrelative fashion. These genes represent a first glimpse into origin-specific molecular targets for HGSOC. Among them, we concentrated on PAX8, a well-known marker of HGSOC and several tumors of Müllerian origin. Of note, its expression and promoter methylation follow the trend existing in normal tissues, suggesting that its expression in tumors could be used as a surrogate lineage tracer, rather than a target for therapy. Nonetheless, evidence shows that the knockdown of PAX8 in HGSOC results in increased apoptosis and reduced proliferation and migration in cancer cell lines [65]. Moreover, the knockdown/overexpression of this gene did not result in tumorigenesis in normal tissues [49, 65], thus suggesting that PAX8 interference could be a potential specific target for HGSOC. Our results build upon this knowledge, allowing to assign PAX8 as a FI-like HGSOC-specific target that could be investigated for improved treatment of this tumor subtype.

## Conclusions

In conclusion, our results demonstrate that both fimbrial and ovarian surface epithelium originate HGSOC in humans, supporting also the adoption of the more appropriate nomenclature high-grade serous tubo-ovarian carcinoma (HGSC), and establish the feasibility of adopting the OriPrint-based classification for a rational stratification of patients. This novel epigenetically guided classification has prognostic relevance and illuminates subtype-specific molecular features to define a rational roadmap towards new therapeutic targets and improved patients’ care.

## Supplementary information

**Additional file 1: Figure S1.** The variance in global DNA methylation does not allow to classify HGSOC according to its cell of origin. **Figure S2.** OriPrint CpGs map preferentially to intergenic regions. **Figure S3.** OriPrint allow stratification of tumor samples. **Figure S4.** The cell of origin is the main determinant of global variance in DNA methylation for HGSOC. **Figure S5.** Diffusion pseudotime on global DNA methylome does not allow to derive an evolutionary line from FI and OSE to tumors. **Figure S6.** Survival curves are consistent after machine learning and show similar results in published cohorts. **Figure S7.** OSE-like tumors show a reduced copy number burden. **Figure S8.** OSE-like tumors present an increased fraction of Memory Resting T cells and M2 macrophages. **Figure S9.** Validation of DEGs between FI-like and OSE-like tumors. **Figure S10.** DNA methylation of promoters in FI-like and OSE-like tumors. (PPTX 6504 kb)

**Additional file 2: Table S1.** Description of the FFPE cohort’s clinical data. **Table S2**. Multivariate analysis on IEO, TCGA, Tothill cohorts. **Table S3**. Summary table of Log Rank Tests performed to determine statistical significance of the differences in survival and progression between FI-like and OSE-like groups in the considered cohorts. **Table S4**. Mutation Frequency in the TCGA cohort stratified as FI-like and OSE-like HGSOC.

## Data Availability

The DNA methylation and RNAseq datasets generated and analyzed during the current study are available in the ArrayExpress repository, under the accession numbers E-MTAB-9608 [66] and E-MTAB-9605 [67], respectively. The code relative to the generation of OriPrint can be found at https://github.com/GiuseppeTestaLab/CellOfOrigin [20].

## Abbreviations

AS: HGSOC from ascites
BRCA1/2: BRCA1 and BRCA2 genes
CUP: Cancer of unknown primary
DDR: DNA damage response
DMS: Differentially methylated sites
FFPE: Formalin-fixed paraffin embedded
FGA: Fraction of the genome altered
FI: Fimbrial epithelium
FI-like: HGSOC derived from fimbrial epithelium
GMM: Gaussian Mixture Model
HGSOC: High-grade serous ovarian cancer
OSE: Ovarian surface epithelium
OSE-like: HGSOC derived from ovarian surface epithelium
STIC: Serous tubal intraepithelial neoplasia
TILs: Tumor-infiltrating lymphocytes
UMAP: Uniform Manifold Approximation
WGCNA: Weighted gene correlation network analysis

## References

[1] Bray F, Ferlay J, Soerjomataram I, Siegel RL, Torre LA, Jemal A (2018). Global cancer statistics 2018: GLOBOCAN estimates of incidence and mortality worldwide for 36 cancers in 185 countries. CA Cancer J Clin.

[2] Reid BM, Permuth JB, Sellers TA (2017). Epidemiology of ovarian cancer: a review. Cancer Biol Med.

[3] Vaughan S, Coward JI, Bast RC, Berchuck A, Berek JS, Brenton JD (2011). Rethinking ovarian cancer: recommendations for improving outcomes. Nat Rev Cancer.

[4] Bowtell DD, Böhm S, Ahmed AA, Aspuria P-J, Bast RC, Beral V (2015). Rethinking ovarian cancer II: reducing mortality from high-grade serous ovarian cancer. Nat Rev Cancer.

[5] Klotz DM, Wimberger P (2017). Cells of origin of ovarian cancer: ovarian surface epithelium or fallopian tube?. Arch Gynecol Obstet.

[6] Ducie J, Dao F, Considine M, Olvera N, Shaw PA, Kurman RJ (2017). Molecular analysis of high-grade serous ovarian carcinoma with and without associated serous tubal intra-epithelial carcinoma. Nat Commun.

[7] Klinkebiel D, Zhang W, Akers SN, Odunsi K, Karpf AR (2016). DNA methylome analyses implicate fallopian tube epithelia as the origin for high-grade serous ovarian cancer. Mol Cancer Res.

[8] Labidi-Galy SI, Papp E, Hallberg D, Niknafs N, Adleff V, Noe M (2017). High grade serous ovarian carcinomas originate in the fallopian tube. Nat Commun.

[9] Eckert MA, Pan S, Hernandez KM, Loth RM, Andrade J, Volchenboum SL (2016). Genomics of ovarian cancer progression reveals diverse metastatic trajectories including intraepithelial metastasis to the fallopian tube. Cancer Discov.

[10] Kulis M, Esteller M (2010). DNA methylation and cancer. Adv Genet.

[11] Kim M, Costello J (2017). DNA methylation: an epigenetic mark of cellular memory. Exp Mol Med.

[12] Moran S, Martínez-Cardús A, Sayols S, Musulén E, Balañá C, Estival-Gonzalez A (2016). Epigenetic profiling to classify cancer of unknown primary: a multicentre, retrospective analysis. Lancet Oncol.

[13] Capper D, Jones DTW, Sill M, Hovestadt V, Schrimpf D, Sturm D (2018). DNA methylation-based classification of central nervous system tumours. Nature..

[14] Francavilla C, Lupia M, Tsafou K, Villa A, Kowalczyk K, Rakownikow Jersie-Christensen R (2017). Phosphoproteomics of primary cells reveals druggable kinase signatures in ovarian cancer. Cell Rep.

[15] Karpf AR. DNA methylome analyses implicate fallopian tube as the tissue of origin for high grade serous ovarian cancer. Gene Expression Omnibus. 2016. https://www.ncbi.nlm.nih.gov/geo/query/acc.cgi?acc=GSE81224. 10.1158/1541-7786.MCR-16-0097 PMC5025356 27259716

[16] Patch A-M, Christie EL, Etemadmoghadam D, Garsed DW, George J, Fereday S (2015). Whole-genome characterization of chemoresistant ovarian cancer. Nature..

[17] The Australian Ovarian Cancer Study Group. Whole genome characterisation of chemoresistant ovarian cancer. Gene Expression Omnibus. 2015. https://www.ncbi.nlm.nih.gov/geo/query/acc.cgi?acc=GSE65820.

[18] Fortin J-P, Triche TJ, Hansen KD (2017). Preprocessing, normalization and integration of the Illumina HumanMethylationEPIC array with minfi. Bioinformatics..

[19] Assenov Y, Müller F, Lutsik P, Walter J, Lengauer T, Bock C (2014). Comprehensive analysis of DNA methylation data with RnBeads. Nat Methods.

[20] Villa CE, Lo Riso P. Lo Riso, Villa et al. 2020. GitHub. https://github.com/GiuseppeTestaLab/CellOfOrigin.

[21] Ritchie ME, Phipson B, Wu D, Hu Y, Law CW, Shi W (2015). limma powers differential expression analyses for RNA-sequencing and microarray studies. Nucleic Acids Res..

[22] Wolf FA, Angerer P, Theis FJ (2018). SCANPY: large-scale single-cell gene expression data analysis. Genome Biol.

[23] Blondel VD, Guillaume J-L, Lambiotte R, Lefebvre E (2008). Fast unfolding of communities in large networks. J Stat Mech.

[24] McInnes L, Healy J, Astels S. hdbscan: Hierarchical density based clustering. JOSS. 2017;2(11):205.

[25] Patro R, Duggal G, Love MI, Irizarry RA, Kingsford C (2017). Salmon provides fast and bias-aware quantification of transcript expression. Nat Methods.

[26] Leek JT. svaseq: removing batch effects and other unwanted noise from sequencing data. Nucleic Acids Res. 2014;42(21):e161. 10.1093/nar/gku864 PMC4245966 25294822

[27] Davidson-Pilon C, Kalderstam J, Kuhn B, Fiore-Gartland A, Moneda L, Zivich P, et al. CamDavidsonPilon/lifelines: v0.14.3. 2018.

[28] Louppe G, Geurts P. Ensembles on random patches. In: Flach PA, De Bie T, Cristianini N, editors. Machine learning and knowledge discovery in databases. Berlin, Heidelberg: Springer Berlin Heidelberg; 2012. 346–361.

[29] Cancer Genome Atlas Research Network (2011). Integrated genomic analyses of ovarian carcinoma. Nature..

[30] The Cancer Genome Atlas Consortium. TCGA-OV. https://portal.gdc.cancer.gov/projects/TCGA-OV.

[31] Tothill RW, Tinker AV, George J, Brown R, Fox SB, Lade S (2008). Novel molecular subtypes of serous and endometrioid ovarian cancer linked to clinical outcome. Clin Cancer Res.

[32] Tothill R, Tinker A, George J, Brown R, Fox S, Johnson D, et al. Expression profile of 285 ovarian tumour samples. Gene Expression Omnibus. https://www.ncbi.nlm.nih.gov/geo/query/acc.cgi?acc=GSE9891.

[33] Becht E, McInnes L, Healy J, Dutertre C-A, Kwok IWH, Ng LG, et al. Dimensionality reduction for visualizing single-cell data using UMAP. Nat Biotechnol. 2018;37:38–44. 10.1038/nbt.4314 30531897

[34] Sandoval J, Heyn H, Moran S, Serra-Musach J, Pujana MA, Bibikova M (2011). Validation of a DNA methylation microarray for 450,000 CpG sites in the human genome. Epigenetics..

[35] Haghverdi L, Buettner F, Theis FJ (2015). Diffusion maps for high-dimensional single-cell analysis of differentiation data. Bioinformatics..

[36] Haghverdi L, Büttner M, Wolf FA, Buettner F, Theis FJ (2016). Diffusion pseudotime robustly reconstructs lineage branching. Nat Methods.

[37] Ho T. The Random Subspace Method for Constructing Decision Forests. IEEE Trans Pattern Anal Mach Intell. 1998;20:832–44.

[38] Despierre E, Moisse M, Yesilyurt B, Sehouli J, Braicu I, Mahner S (2014). Somatic copy number alterations predict response to platinum therapy in epithelial ovarian cancer. Gynecol Oncol.

[39] Leong HS, Galletta L, Etemadmoghadam D, George J, Australian Ovarian Cancer Study, Köbel M, et al. Efficient molecular subtype classification of high-grade serous ovarian cancer. J Pathol 2015;236(3):272–277. 10.1002/path.4536 25810134

[40] Newman AM, Liu CL, Green MR, Gentles AJ, Feng W, Xu Y (2015). Robust enumeration of cell subsets from tissue expression profiles. Nat Methods.

[41] Brown JM, Recht L, Strober S (2017). The promise of targeting macrophages in cancer therapy. Clin Cancer Res.

[42] Allen F, Bobanga ID, Rauhe P, Barkauskas D, Teich N, Tong C, et al. CCL3 augments tumor rejection and enhances CD8+ T cell infiltration through NK and CD103+ dendritic cell recruitment via IFNγ. Oncoimmunology. 2018;7(3):e1393598. 10.1080/2162402X.2017.1393598 PMC5790335 29399390

[43] Ouyang W, O’Garra A (2019). IL-10 family cytokines IL-10 and IL-22: from basic science to clinical translation. Immunity..

[44] Yang L, Pang Y, Moses HL (2010). TGF-beta and immune cells: an important regulatory axis in the tumor microenvironment and progression. Trends Immunol.

[45] Tsukamoto H, Fujieda K, Miyashita A, Fukushima S, Ikeda T, Kubo Y (2018). Combined blockade of IL6 and PD-1/PD-L1 signaling abrogates mutual regulation of their immunosuppressive effects in the tumor microenvironment. Cancer Res.

[46] Susek KH, Karvouni M, Alici E, Lundqvist A (2018). The role of CXC chemokine receptors 1-4 on immune cells in the tumor microenvironment. Front Immunol.

[47] Baker KJ, Houston A, Brint E (2019). IL-1 family members in cancer; two sides to every story. Front Immunol.

[48] Li Y-L, Zhao H, Ren X-B (2016). Relationship of VEGF/VEGFR with immune and cancer cells: staggering or forward?. Cancer Biol Med..

[49] Hardy LR, Salvi A, Burdette JE. UnPAXing the divergent roles of PAX2 and PAX8 in high-grade serous ovarian cancer. Cancers (Basel). 2018;10(8):262. 10.3390/cancers10080262 PMC6115736 30096791

[50] Adler EK, Corona RI, Lee JM, Rodriguez-Malave N, Mhawech-Fauceglia P, Sowter H (2017). The PAX8 cistrome in epithelial ovarian cancer. Oncotarget..

[51] Fathalla MF (2013). Incessant ovulation and ovarian cancer - a hypothesis re-visited. Facts Views Vis Obgyn.

[52] Kim J, Coffey DM, Creighton CJ, Yu Z, Hawkins SM, Matzuk MM (2012). High-grade serous ovarian cancer arises from fallopian tube in a mouse model. Proc Natl Acad Sci U S A.

[53] Kim J, Coffey DM, Ma L, Matzuk MM (2015). The ovary is an alternative site of origin for high-grade serous ovarian cancer in mice. Endocrinology..

[54] Perets R, Wyant GA, Muto KW, Bijron JG, Poole BB, Chin KT (2013). Transformation of the fallopian tube secretory epithelium leads to high-grade serous ovarian cancer in Brca;Tp53;Pten models. Cancer Cell.

[55] Zhang S, Dolgalev I, Zhang T, Ran H, Levine DA, Neel BG (2019). Both fallopian tube and ovarian surface epithelium are cells-of-origin for high-grade serous ovarian carcinoma. Nat Commun.

[56] Lawrenson K, Fonseca MAS, Liu AY, Segato Dezem F, Lee JM, Lin X (2019). A study of high-grade serous ovarian cancer origins implicates the SOX18 transcription factor in tumor development. Cell Rep..

[57] Hao D, Li J, Jia S, Meng Y, Zhang C, Wang L (2017). Integrated analysis reveals tubal- and ovarian-originated serous ovarian cancer and predicts differential therapeutic responses. Clin Cancer Res.

[58] Coscia F, Watters KM, Curtis M, Eckert MA, Chiang CY, Tyanova S (2016). Integrative proteomic profiling of ovarian cancer cell lines reveals precursor cell associated proteins and functional status. Nat Commun.

[59] Cieślik M, Chinnaiyan AM (2018). Cancer transcriptome profiling at the juncture of clinical translation. Nat Rev Genet.

[60] Lord CJ, Ashworth A (2016). BRCAness revisited. Nat Rev Cancer.

[61] Lõhmussaar K, Kopper O, Korving J, Begthel H, Vreuls CPH, van Es JH (2020). Assessing the origin of high-grade serous ovarian cancer using CRISPR-modification of mouse organoids. Nat Commun.

[62] Goode EL, Block MS, Kalli KR, Vierkant RA, Chen W, Ovarian Tumor Tissue Analysis (OTTA) Consortium (2017). Dose-response association of CD8+ tumor-infiltrating lymphocytes and survival time in high-grade serous ovarian cancer. JAMA Oncol.

[63] Fritz JM, Lenardo MJ (2019). Development of immune checkpoint therapy for cancer. J Exp Med.

[64] Chabanon RM, Pedrero M, Lefebvre C, Marabelle A, Soria J-C, Postel-Vinay S (2016). Mutational landscape and sensitivity to immune checkpoint blockers. Clin Cancer Res.

[65] Rodgers LH, Ó hAinmhire E, Young AN, Burdette JE. Loss of PAX8 in high-grade serous ovarian cancer reduces cell survival despite unique modes of action in the fallopian tube and ovarian surface epithelium. Oncotarget. 2016;7(22):32785–32795. 10.18632/oncotarget.9051 PMC5078051 27129161

[66] Villa CE, Lo Riso P. DNA methylation profiling of fimbrial epithelium, ovarian surface epithelium, solid and ascitis-derived high grade serous ovarian cancer 2D cultures. ArrayExpress. https://www.ebi.ac.uk/arrayexpress/experiments/E-MTAB-9608/.

[67] Villa CE, Lo Riso P. RNA-seq of FFPE-macrodissected HGSOC tissues and solid and ascites-derived HGSOC 2D cultures. ArrayExpress. https://www.ebi.ac.uk/arrayexpress/experiments/E-MTAB-9605/.

