# Supplementary material for: A cell-of-origin epigenetic tracer reveals clinically distinct subtypes of high-grade serous ovarian cancer
Source: Genome Med. 2020 Oct 30;12:94. doi: 10.1186/s13073-020-00786-7 (PMC7597028; doi:10.1186/s13073-020-00786-7)
Supplement: Supplementary file 1 — Additional file 1: Figure S1. The variance in global DNA methylation does not allow to classify HGSOC according to its cell of origin. Figure S2. OriPrint CpGs map preferentially to intergenic regions. Figure S3. OriPrint allow stratification of tumor samples. Figure S4. The cell of origin is the main determinant of global variance in DNA methylation for HGSOC. Figure S5. Diffusion pseudotime on global DNA methylome does not allow to derive an evolutionary line from FI and OSE to tumors. Figure S6. Survival curves are consistent after machine learning and show similar results in published cohorts. Figure S7. OSE-like tumors show a reduced copy number burden. Figure S8. OSE-like tumors present an increased fraction of Memory Resting T cells and M2 macrophages. Figure S9. Validation of DEGs between FI-like and OSE-like tumors. Figure S10. DNA methylation of promoters in FI-like and OSE-like tumors. (PPTX 6504 kb) [file 13073_2020_786_MOESM1_ESM.pptx]

## Slide 1
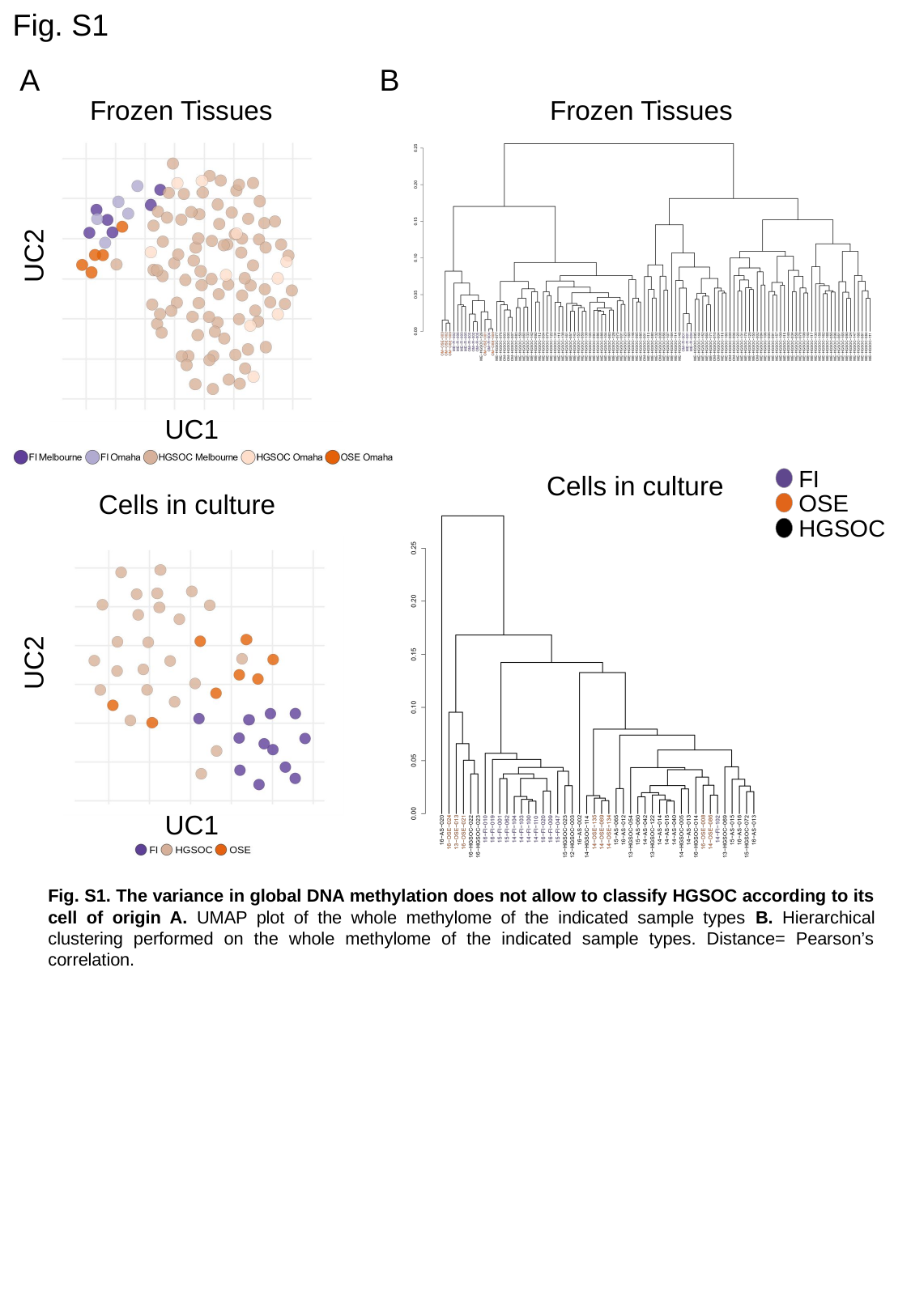

Fig. S1
A
B
Frozen Tissues
UC2
UC1
Frozen Tissues
FI
Cells in culture
OSE
HGSOC
Cells in culture
UC2
UC1
Fig. S1. The variance in global DNA methylation does not allow to classify HGSOC according to its cell of origin A. UMAP plot of the whole methylome of the indicated sample types B. Hierarchical clustering performed on the whole methylome of the indicated sample types. Distance= Pearson’s correlation.

## Slide 2
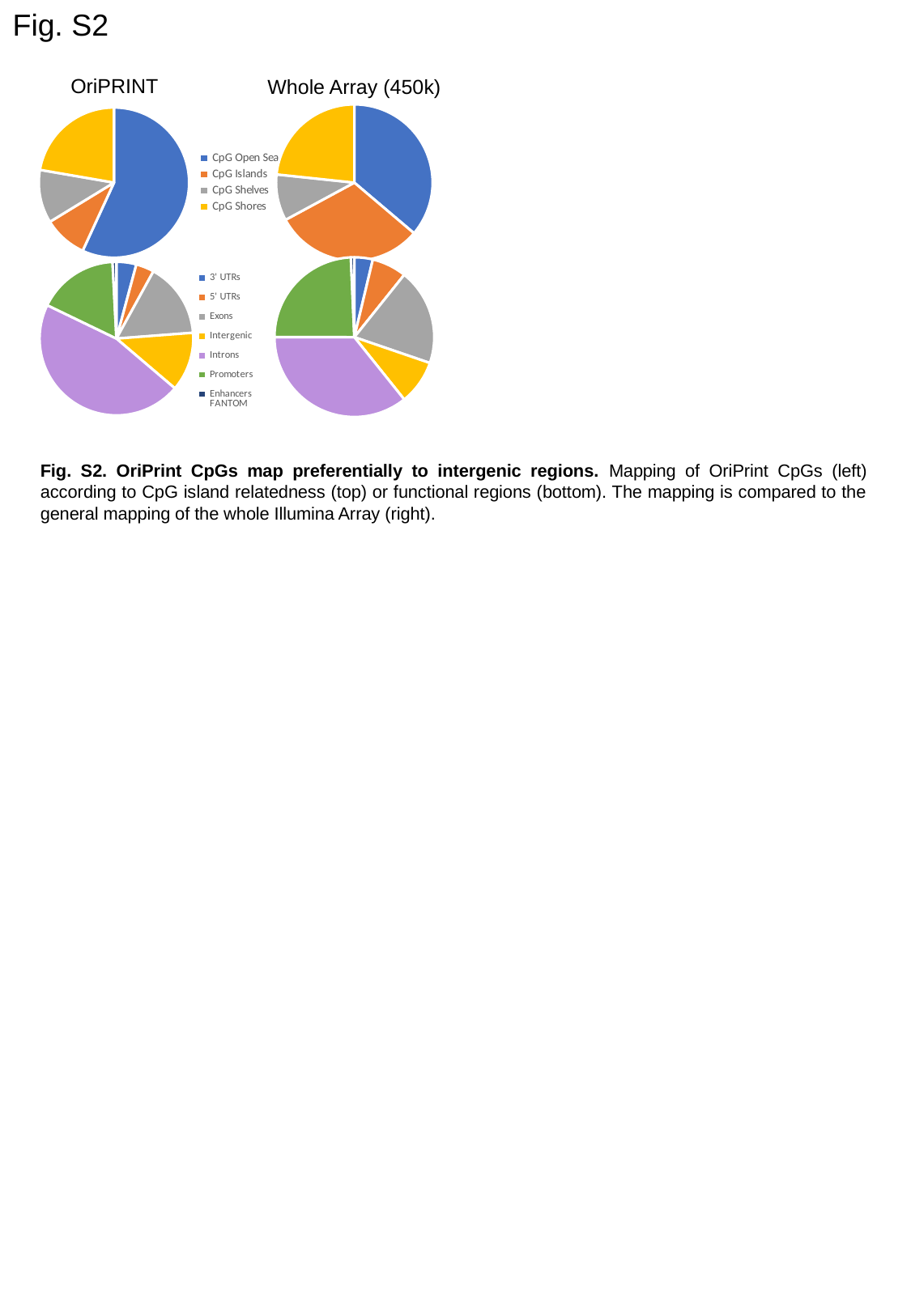

Fig. S2
OriPRINT
Whole Array (450k)
### Chart
| Category | 450K array |
|---|---|
| CpG Open Sea | 173931.0 |
| CpG Islands | 148464.0 |
| CpG Shelves | 46021.0 |
| CpG Shores | 112057.0 |
### Chart
| Category | 13926 DMS |
|---|---|
| CpG Open Sea | 7900.0 |
| CpG Islands | 1308.0 |
| CpG Shelves | 1594.0 |
| CpG Shores | 3096.0 |
### Chart
| Category | 450K array |
|---|---|
| 3' UTRs | 26854.0 |
| 5' UTRs | 50419.0 |
| Exons | 140391.0 |
| Intergenic | 64720.0 |
| Introns | 257222.0 |
| Promoters | 174731.0 |
| Enhancers FANTOM | 4988.0 |
### Chart
| Category | 13926 DMS |
|---|---|
| 3' UTRs | 779.0 |
| 5' UTRs | 714.0 |
| Exons | 2944.0 |
| Intergenic | 2320.0 |
| Introns | 8567.0 |
| Promoters | 3176.0 |
| Enhancers FANTOM | 152.0 |Fig. S2. OriPrint CpGs map preferentially to intergenic regions. Mapping of OriPrint CpGs (left) according to CpG island relatedness (top) or functional regions (bottom). The mapping is compared to the general mapping of the whole Illumina Array (right).

## Slide 3
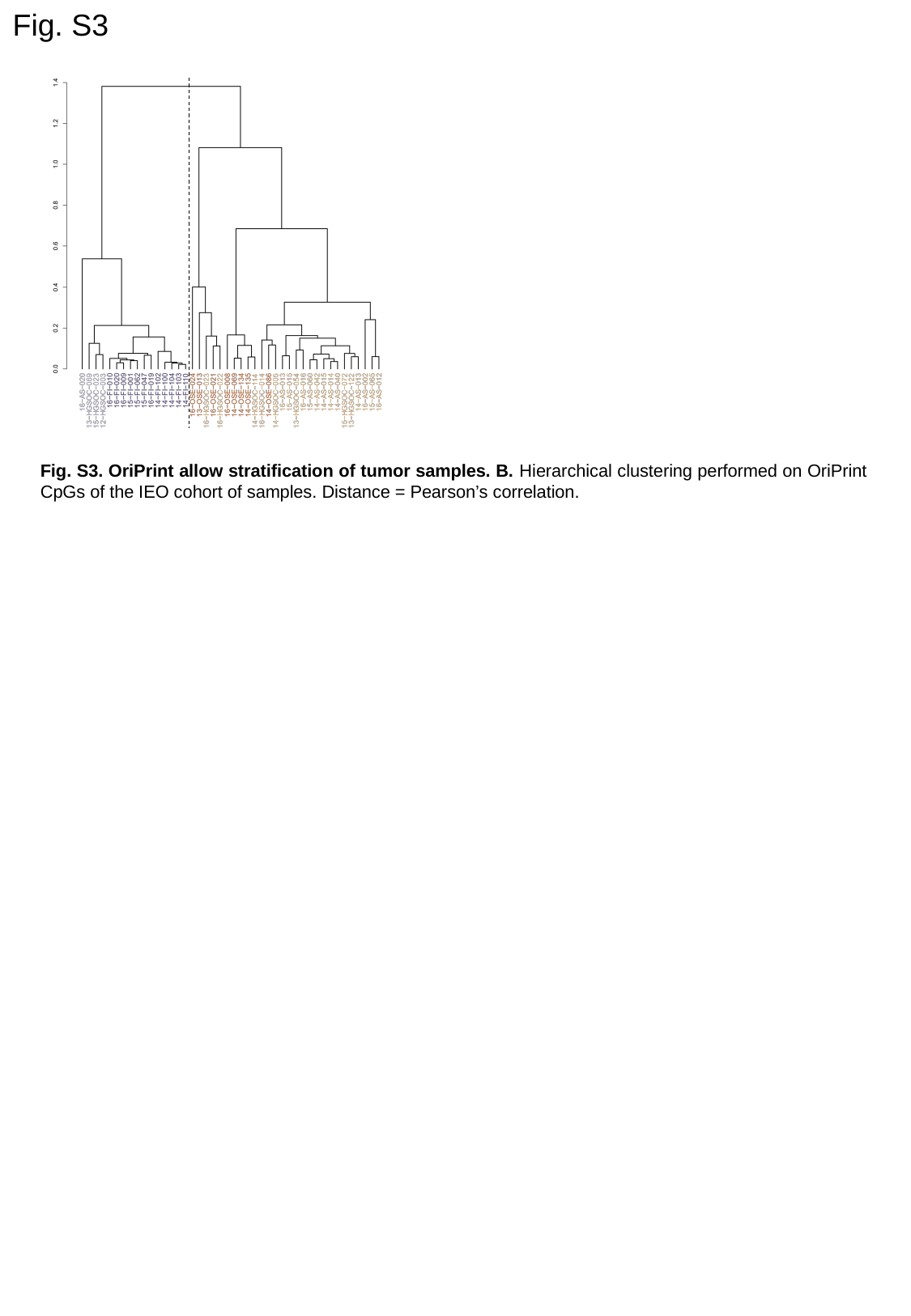

Fig. S3
Fig. S3. OriPrint allow stratification of tumor samples. B. Hierarchical clustering performed on OriPrint CpGs of the IEO cohort of samples. Distance = Pearson’s correlation.

## Slide 4
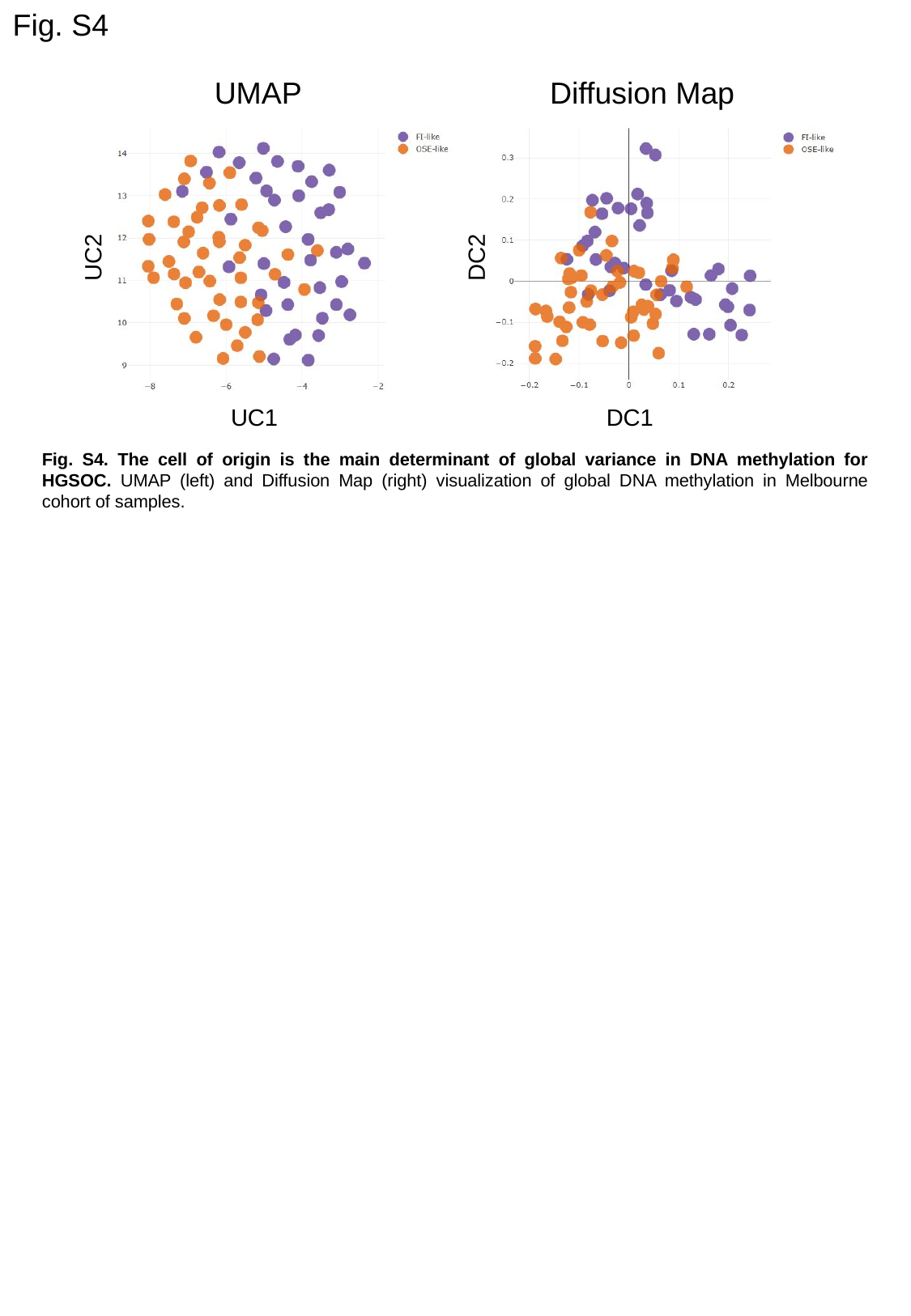

Fig. S4
UMAP
Diffusion Map
UC2
DC2
UC1
DC1
Fig. S4. The cell of origin is the main determinant of global variance in DNA methylation for HGSOC. UMAP (left) and Diffusion Map (right) visualization of global DNA methylation in Melbourne cohort of samples.

## Slide 5
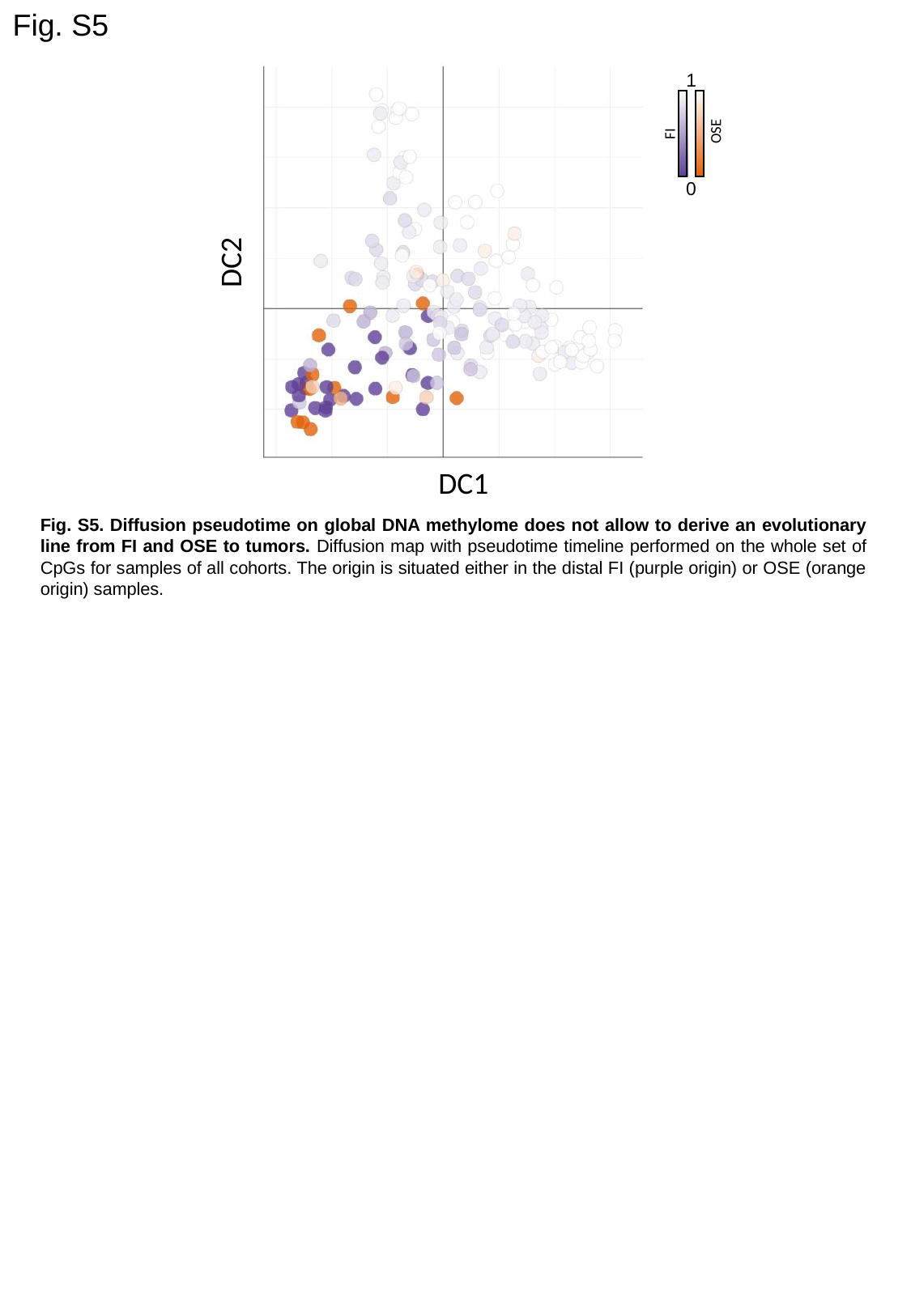

Fig. S5
1
OSE
FI
0
DC2
DC1
Fig. S5. Diffusion pseudotime on global DNA methylome does not allow to derive an evolutionary line from FI and OSE to tumors. Diffusion map with pseudotime timeline performed on the whole set of CpGs for samples of all cohorts. The origin is situated either in the distal FI (purple origin) or OSE (orange origin) samples.

## Slide 6
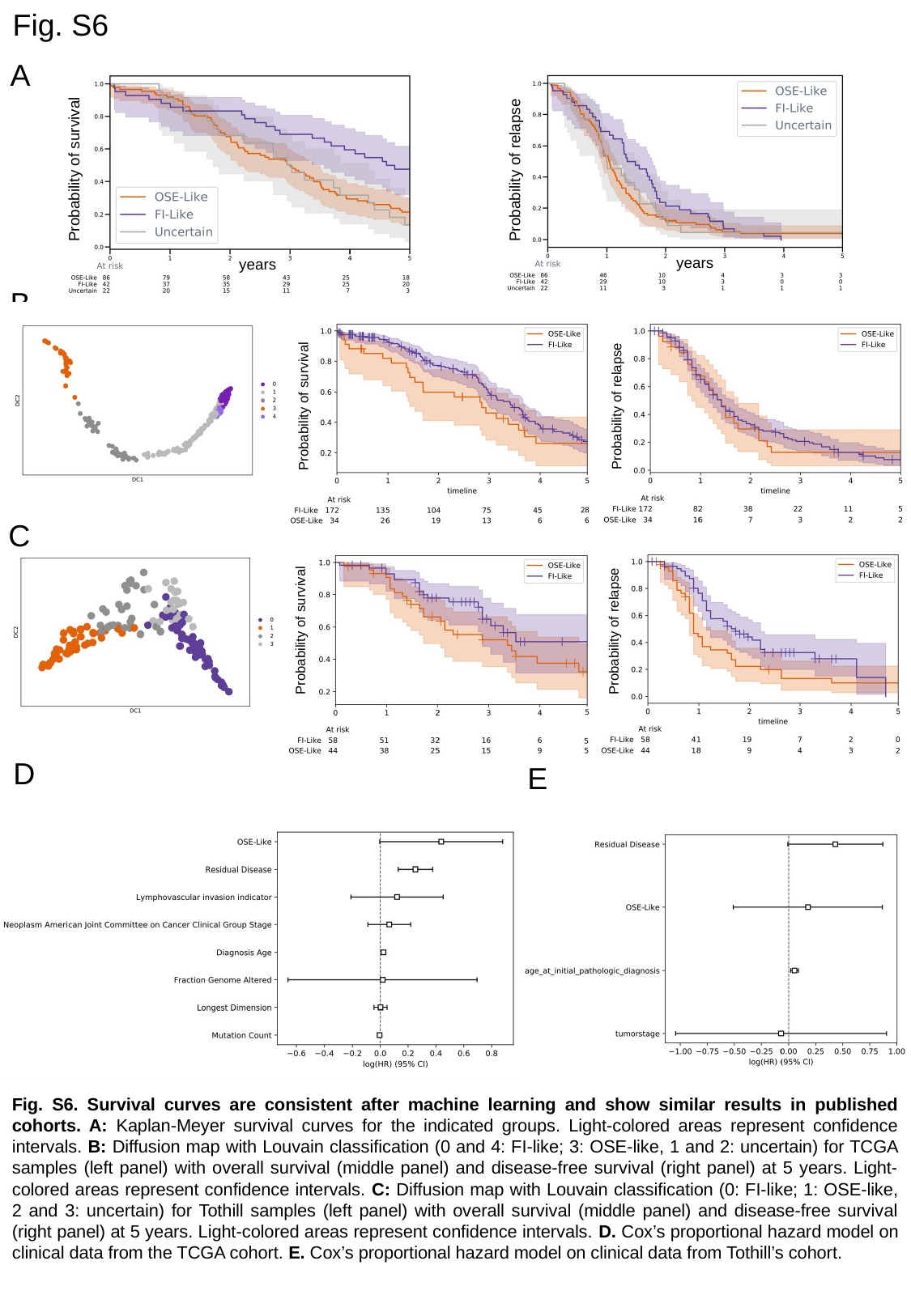

Fig. S6
A
Probability of survival
Probability of relapse
years
years
B
Probability of relapse
Probability of survival
C
Probability of survival
Probability of relapse
D
E
Fig. S6. Survival curves are consistent after machine learning and show similar results in published cohorts. A: Kaplan-Meyer survival curves for the indicated groups. Light-colored areas represent confidence intervals. B: Diffusion map with Louvain classification (0 and 4: FI-like; 3: OSE-like, 1 and 2: uncertain) for TCGA samples (left panel) with overall survival (middle panel) and disease-free survival (right panel) at 5 years. Light-colored areas represent confidence intervals. C: Diffusion map with Louvain classification (0: FI-like; 1: OSE-like, 2 and 3: uncertain) for Tothill samples (left panel) with overall survival (middle panel) and disease-free survival (right panel) at 5 years. Light-colored areas represent confidence intervals. D. Cox’s proportional hazard model on clinical data from the TCGA cohort. E. Cox’s proportional hazard model on clinical data from Tothill’s cohort.

## Slide 7
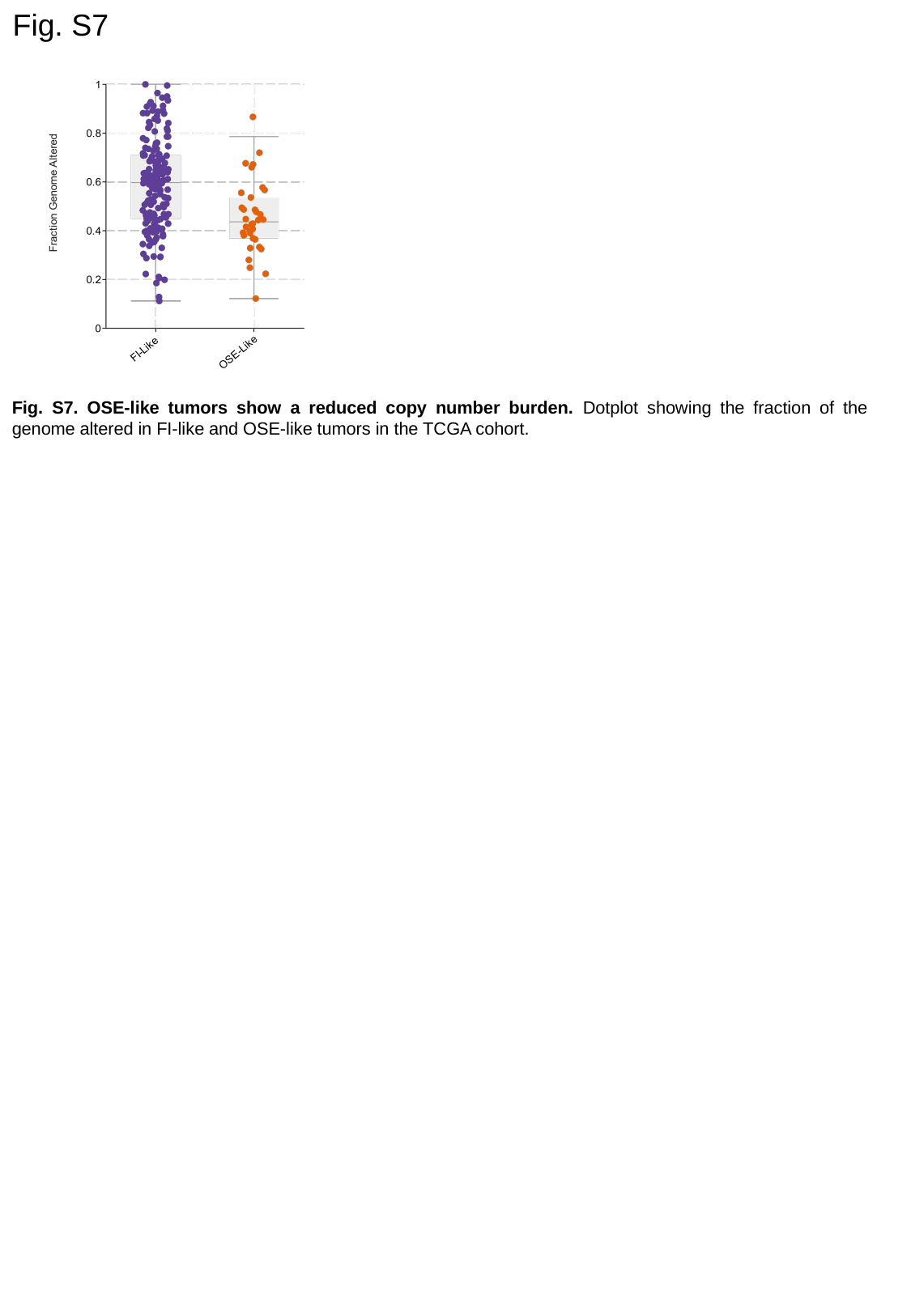

Fig. S7
Fig. S7. OSE-like tumors show a reduced copy number burden. Dotplot showing the fraction of the genome altered in FI-like and OSE-like tumors in the TCGA cohort.

## Slide 8
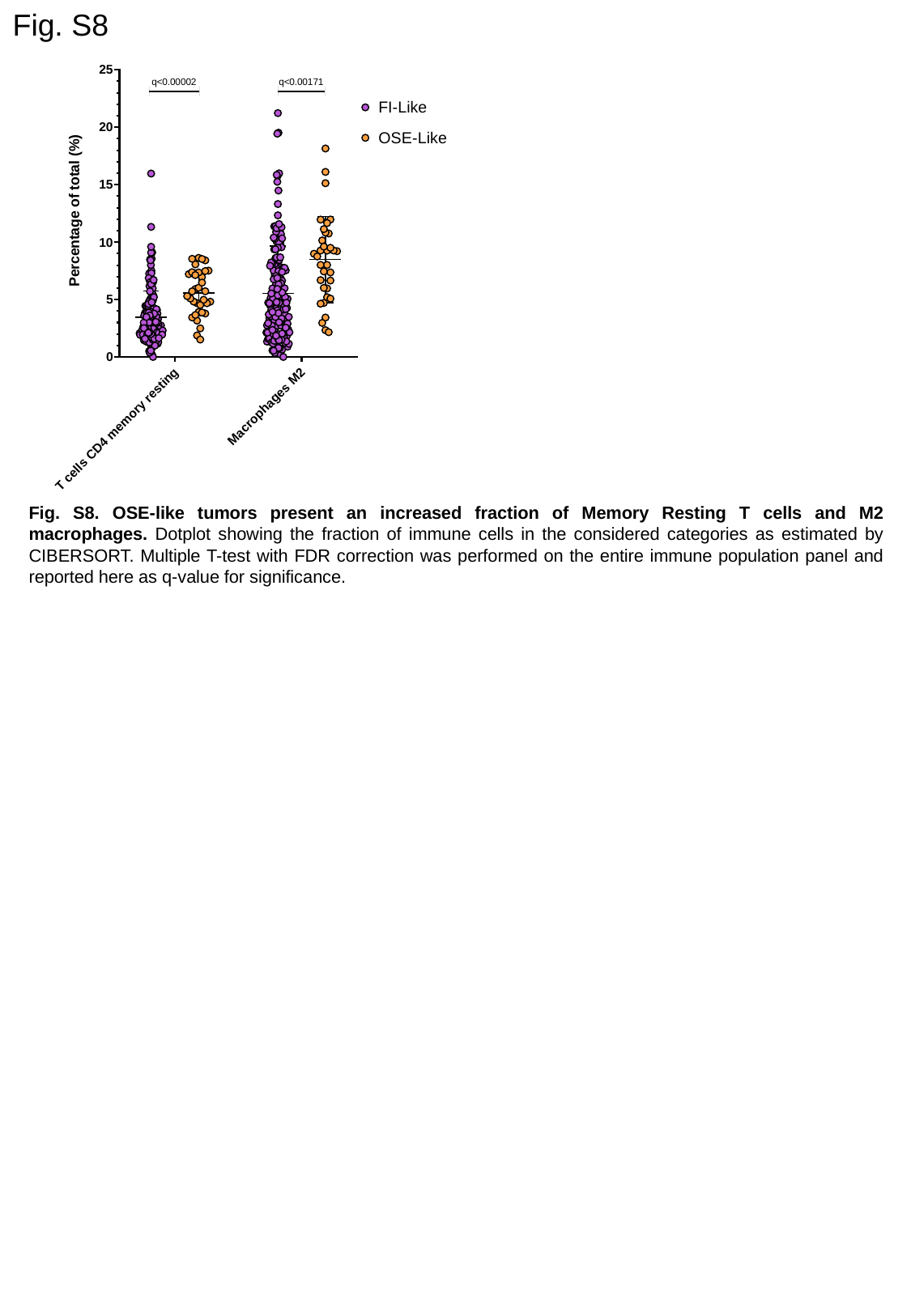

Fig. S8
Fig. S8. OSE-like tumors present an increased fraction of Memory Resting T cells and M2 macrophages. Dotplot showing the fraction of immune cells in the considered categories as estimated by CIBERSORT. Multiple T-test with FDR correction was performed on the entire immune population panel and reported here as q-value for significance.

## Slide 9
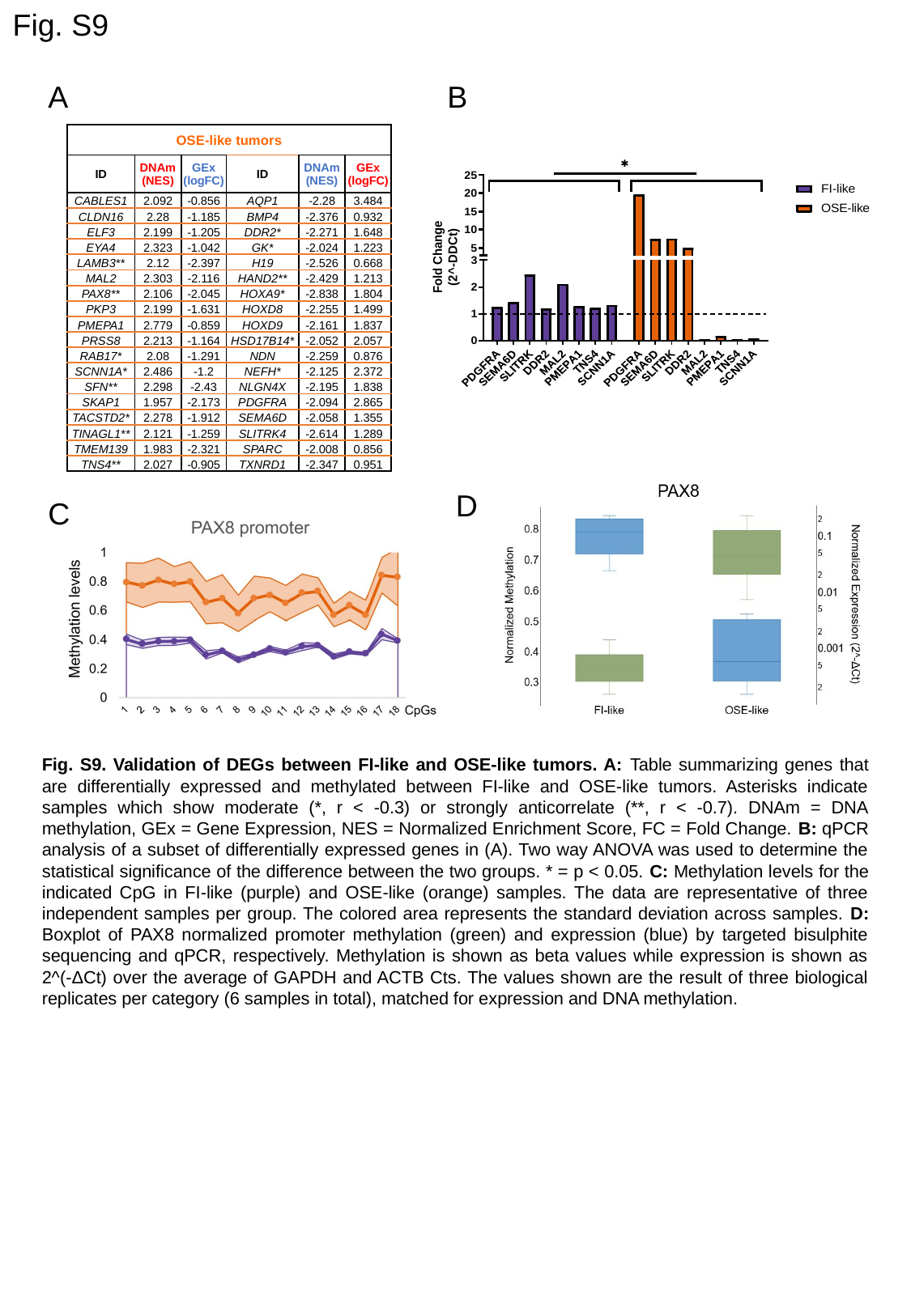

Fig. S9
A
B
| OSE-like tumors | | | | | |
| --- | --- | --- | --- | --- | --- |
| ID | DNAm (NES) | GEx (logFC) | ID | DNAm (NES) | GEx (logFC) |
| CABLES1 | 2.092 | -0.856 | AQP1 | -2.28 | 3.484 |
| CLDN16 | 2.28 | -1.185 | BMP4 | -2.376 | 0.932 |
| ELF3 | 2.199 | -1.205 | DDR2\* | -2.271 | 1.648 |
| EYA4 | 2.323 | -1.042 | GK\* | -2.024 | 1.223 |
| LAMB3\*\* | 2.12 | -2.397 | H19 | -2.526 | 0.668 |
| MAL2 | 2.303 | -2.116 | HAND2\*\* | -2.429 | 1.213 |
| PAX8\*\* | 2.106 | -2.045 | HOXA9\* | -2.838 | 1.804 |
| PKP3 | 2.199 | -1.631 | HOXD8 | -2.255 | 1.499 |
| PMEPA1 | 2.779 | -0.859 | HOXD9 | -2.161 | 1.837 |
| PRSS8 | 2.213 | -1.164 | HSD17B14\* | -2.052 | 2.057 |
| RAB17\* | 2.08 | -1.291 | NDN | -2.259 | 0.876 |
| SCNN1A\* | 2.486 | -1.2 | NEFH\* | -2.125 | 2.372 |
| SFN\*\* | 2.298 | -2.43 | NLGN4X | -2.195 | 1.838 |
| SKAP1 | 1.957 | -2.173 | PDGFRA | -2.094 | 2.865 |
| TACSTD2\* | 2.278 | -1.912 | SEMA6D | -2.058 | 1.355 |
| TINAGL1\*\* | 2.121 | -1.259 | SLITRK4 | -2.614 | 1.289 |
| TMEM139 | 1.983 | -2.321 | SPARC | -2.008 | 0.856 |
| TNS4\*\* | 2.027 | -0.905 | TXNRD1 | -2.347 | 0.951 |
D
C
Fig. S9. Validation of DEGs between FI-like and OSE-like tumors. A: Table summarizing genes that are differentially expressed and methylated between FI-like and OSE-like tumors. Asterisks indicate samples which show moderate (*, r < -0.3) or strongly anticorrelate (**, r < -0.7). DNAm = DNA methylation, GEx = Gene Expression, NES = Normalized Enrichment Score, FC = Fold Change. B: qPCR analysis of a subset of differentially expressed genes in (A). Two way ANOVA was used to determine the statistical significance of the difference between the two groups. * = p < 0.05. C: Methylation levels for the indicated CpG in FI-like (purple) and OSE-like (orange) samples. The data are representative of three independent samples per group. The colored area represents the standard deviation across samples. D: Boxplot of PAX8 normalized promoter methylation (green) and expression (blue) by targeted bisulphite sequencing and qPCR, respectively. Methylation is shown as beta values while expression is shown as 2^(-ΔCt) over the average of GAPDH and ACTB Cts. The values shown are the result of three biological replicates per category (6 samples in total), matched for expression and DNA methylation.

## Slide 10
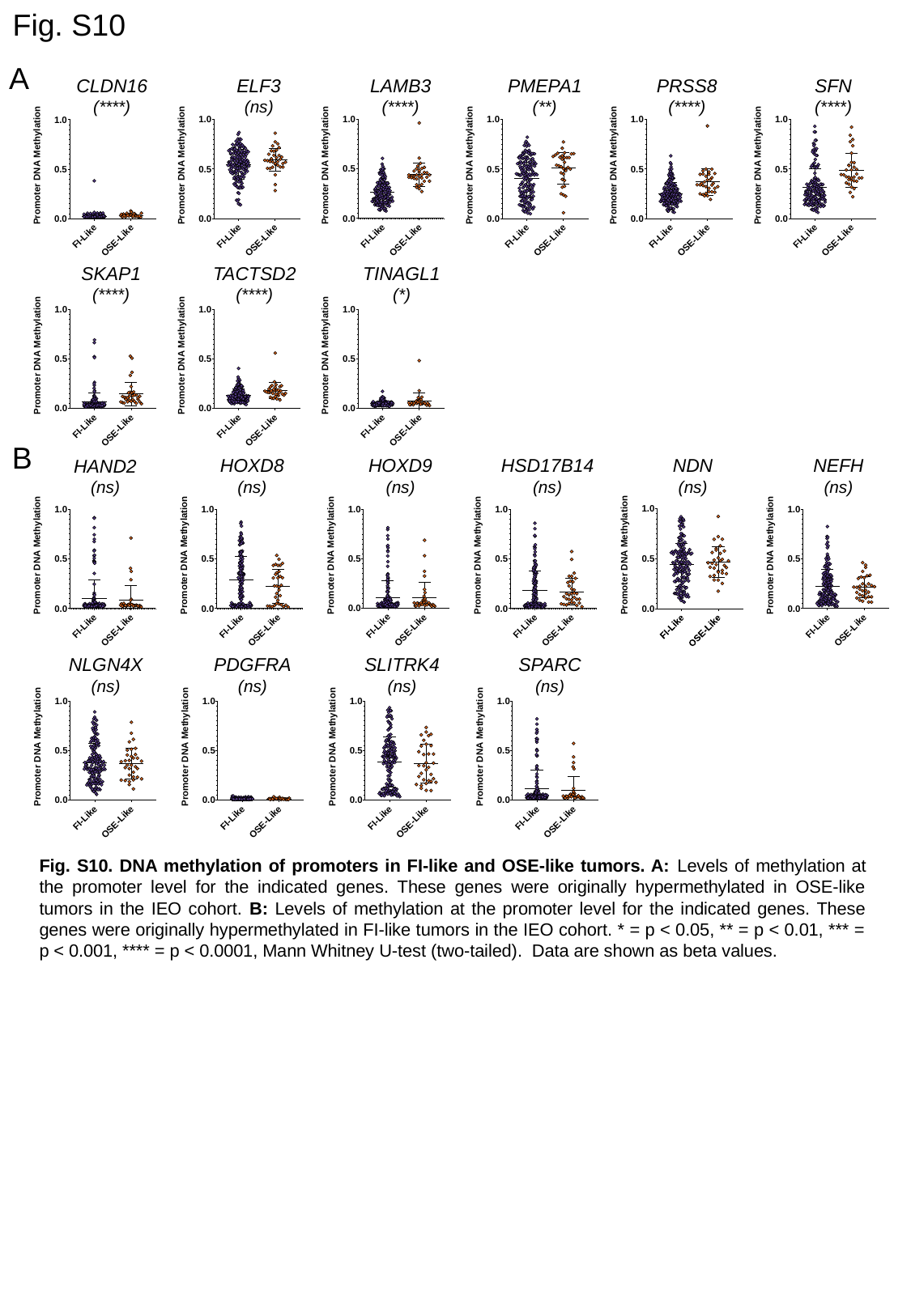

Fig. S10
A
CLDN16
(****)
ELF3
(ns)
LAMB3
(****)
PMEPA1
(**)
PRSS8
(****)
SFN
(****)
SKAP1
(****)
TACTSD2
(****)
TINAGL1
(*)
B
HAND2
(ns)
HOXD8
(ns)
HOXD9
(ns)
HSD17B14
(ns)
NDN
(ns)
NEFH
(ns)
NLGN4X
(ns)
PDGFRA
(ns)
SLITRK4
(ns)
SPARC
(ns)
Fig. S10. DNA methylation of promoters in FI-like and OSE-like tumors. A: Levels of methylation at the promoter level for the indicated genes. These genes were originally hypermethylated in OSE-like tumors in the IEO cohort. B: Levels of methylation at the promoter level for the indicated genes. These genes were originally hypermethylated in FI-like tumors in the IEO cohort. * = p < 0.05, ** = p < 0.01, *** = p < 0.001, **** = p < 0.0001, Mann Whitney U-test (two-tailed). Data are shown as beta values.
